# LFQRatio: A Normalization Method to Decipher Quantitative Proteome Changes in Microbial Coculture Systems

**DOI:** 10.1021/acs.jproteome.3c00714

**Published:** 2024-02-14

**Authors:** Mengxun Shi, Caroline A. Evans, Josie L. McQuillan, Josselin Noirel, Jagroop Pandhal

**Affiliations:** † Department of Chemical and Biological Engineering, The University of Sheffield, Mappin Street, Sheffield S1 3JD, U.K.; ‡ GBCM Laboratory (EA7528), Conservatoire National des Arts et Métiers, HESAM Université, 2 rue Conté, Paris 75003, France

**Keywords:** microbial coculture, quantitative proteomics, label-free quantification, *Synechococcus*, *Azotobacter*

## Abstract

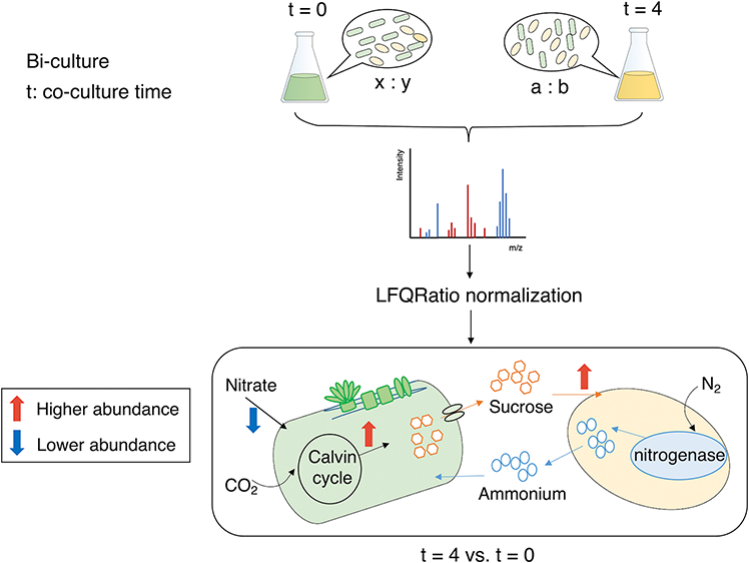

The value of synthetic microbial communities in biotechnology is gaining traction due to their ability to undertake more complex metabolic tasks than monocultures. However, a thorough understanding of strain interactions, productivity, and stability is often required to optimize growth and scale up cultivation. Quantitative proteomics can provide valuable insights into how microbial strains adapt to changing conditions in biomanufacturing. However, current workflows and methodologies are not suitable for simple artificial coculture systems where strain ratios are dynamic. Here, we established a workflow for coculture proteomics using an exemplar system containing two members, *Azotobacter vinelandii* and *Synechococcus elongatus*. Factors affecting the quantitative accuracy of coculture proteomics were investigated, including peptide physicochemical characteristics such as molecular weight, isoelectric point, hydrophobicity, and dynamic range as well as factors relating to protein identification such as varying proteome size and shared peptides between species. Different quantification methods based on spectral counts and intensity were evaluated at the protein and cell level. We propose a new normalization method, named “LFQRatio”, to reflect the relative contributions of two distinct cell types emerging from cell ratio changes during cocultivation. LFQRatio can be applied to real coculture proteomics experiments, providing accurate insights into quantitative proteome changes in each strain.

## Introduction

There is growing interest in a variety of fields in the creation of synthetic microbial consortia. This can be for the fundamental understanding of how microbes interact,^1^ microbial evolution studies,^2,3^ or biotechnology purposes.^4−6^ Quantitative proteomics provides a powerful tool to interrogate how these microbes interact and their relative metabolic status under different conditions.

Although proteomics workflows to quantify protein abundance changes in different conditions (e.g., nutrient limitation, light and dark cycles, etc.) are widely available for axenic (pure) microbial cultures,^7,8^ applying these common workflows to synthetic cocultures, i.e., when several strain types are cultivated together, presents certain challenges. This is particularly relevant when conditions with highly variable cell-type ratios are compared, as this will cause large differences in protein abundance between samples. Advanced software algorithms can deal with some systematic biases among samples, e.g., samples processed on different days or with different MS performances,^9,10^ as well as for differences in protein extraction efficiency among coculture cell types; however, they produce unreliable quantification data when analyzing samples with large cell number differences,^11^ such as comparing monocultures and cocultures, or different coculture time points. In metaproteomics experiments, species abundances are often quantified and considered in interpreting the findings of the data. Quantifying cell numbers can be undertaken using well-established methods such as 16S rRNA gene amplicon sequencing and fluorescence in situ hybridization. Biomass abundances can even be calculated using mass spectra, and methodologies have been demonstrated to quantify organisms in saliva from multiple individuals and microbial mats from two alkaline soda lakes.^11,12^ However, a method to account for the two distinct protein populations present in samples for quantitative proteomics analysis remains to be established.

Key considerations for such an approach are the physicochemical characteristics of consortium member proteomes, such as the range of isoelectric points (pI),^13^ molecular weight (*M*_w_),^14^ and hydrophobicity,^15^ as well as the dynamic range distribution of protein abundances within the proteome,^16^ which must be considered when analyzing mixed-strain proteomic samples, as they will affect protein extraction efficiency, peptide ionization in the mass spectrometer and overall proteome identification, among other factors. There are also protein database-related challenges, for bioinformatics analysis including variable proteome sizes^17^ and shared peptides between the strains that must be considered in terms of protein assignment and relative quantification.^18^

Previous proteomic studies of synthetic cocultures often compare biculture (two-member strains) to monoculture (single strain), and hence, physicochemical and bioinformatics factors that affect quantification do not need to be considered.^18^ If the aim is to infer metabolic changes in each strain in the coculture, there are cultivation strategies that can overcome technical challenges. One option is spatial separation of the strains, for example, in growth chambers separated by a semipermeable membrane that allows metabolite exchange but not cell mixing.^19,20^ Standard proteomics workflows could then be applied to each sample separately. Furthermore, if strains are sufficiently different in size or fluorescence, they can be separated by flow cytometry and cell sorting^21,22^ or differential centrifugation,^23^ although this process has its inherent limitations: (a) overlap in the size or fluorescence distributions of the strains and (b) changing cell cultivation volumes,^24^ which could affect metabolism and therefore the proteome.^25^ When cultivated together in the same vessel, the presence of more than one proteome has been shown to impact the detection of proteins from each species.^26,27^

Quantification in proteomics has largely moved to label-free methods due to the rapid improvement in the sensitivity of liquid chromatography (LC), mass spectrometry hardware, and the accuracy of proteomics data analysis tools.^28^ Label-free quantification (LFQ) has the benefit of cost savings, less stringent chemistry requirements in extraction buffers, and no limitation of sample numbers, compared with label-based methods.^29^ LFQ is widely used to analyze global proteome changes in different biological conditions, allowing for quantification of thousands of proteins to be determined using the “total protein approach”.^30,31^ There are two main categories of relative quantification methods for label-free proteomics. The first is based on spectral counting data generated during protein identification such as peptide counts and peptide-to-spectrum matches (PSMs). The second is based on spectral intensity, such as peptide peak heights and peak areas.^32,33^ Spectral counting-based methods have gained widespread use due to their ease of implementation;^34^ however, they are significantly affected by the dynamic exclusion settings of the mass spectrometer, which can obscure the relationship between the detected number of counts and protein abundance, especially for lower abundance proteins. Intensity-based methods can be applied as an alternative, although MS2 methods are not always accurate, as peptide fragmentation sometimes does not occur at the apex of the elution peak.^35^ In addition, some methods combining both approaches have been developed, such as ProPCA.^36^

Here, our aim was to develop an LFQ proteomics workflow to temporally quantify the proteomes of a coculture system. We focused on a coculture of *Synochococcus elongatus* cscB/SPS^37^ and *Azotobacter vinelandii* ΔnifL,^38^ herein referred to as *S. elongatus* and *A. vinelandii*, respectively. *S. elongatus* is a model freshwater cyanobacterium with the capacity to produce biotechnologically relevant chemicals and biofuels, including acetone,^39^ glycerol,^40^ fatty acids,^41^ etc. The strain used in this study was engineered to secrete sucrose, using carbon sourced from CO_2_ fixation and hence has generated significant interest in the microbial biotechnology community.^37,42^*A. vinelandii* is an obligate aerobic nitrogen-fixing bacterium that has been used as a model to study nitrogen fixation and siderophore production.^43^ The strain used here can secrete ammonium. These strains were selected as they are engineered to require each other for cross-feeding nitrogen and carbon nutrients (as sucrose and ammonium, respectively), and hence, there are predictable changes in the functional proteome over time in coculture. They are also of interest biotechnologically. Although these strains were successfully combined and shown to survive without organic forms of carbon and nitrogen in the medium,^44^ they exhibited signs of physiological stress and ratios of each cell type varied drastically over time. A quantitative proteomics analysis could be used to characterize metabolic constraints from the perspective of both strains, making them an ideal model coculture for this study.

To develop a quantitative proteomics analytical workflow for mixed microbial cultures, we compared the proteome physiochemical characteristics, proteome sizes, and shared peptides between *S. elongatus* and *A. vinelandii*. We then generated a series of mixed samples containing known ratios of (1) each cell type or (2) protein extracts from each strain and analyzed the variability in estimated protein abundance for the sample series. Estimation was assessed using six metrics based on spectral counts [PSMs, unique peptides, and normalized spectral abundance factor (NSAF)] and spectral intensity (intensity, iBAQ intensity, and LFQ intensity) at both the protein level and cell level. Guided by these data, we developed a novel normalization method, “LFQRatio”, which, unlike the analytical methods currently available, can accommodate large differences in cell number ratios observed between coculture conditions. LFQRatio factors in the LFQ intensity ratio of each protein and the total protein intensity to generate accurate label-free proteome quantification data for both strains within the microbial coculture.

## Experimental Procedures

### Cell Cultivation

For the synthetic cell mix cultures, *S. elongatus* culture was grown in BG11 medium,^45^ pH 8.0, at 30 °C with agitation at 120 rpm and light intensity at 120 μE m^–2^ s^–1^. *A. vinelandii* was cultivated in Burk’s medium^46^ at 30 °C with agitation at 150 rpm. *S. elongatus* and *A. vinelandii* cells were grown to the exponential phase, the seventh day and third day, respectively, and collected for the sample mixes.

*S. elongatus* and *A. vinelandii* cocultures were grown in an optimized medium based on BG11 medium and Burk’s medium (Table S1) with data generated from three biological replicates. Briefly, after growing to the early mid-log phase for 7 days, *S. elongatus* was washed with PBS buffer and inoculated into coculture medium with 0.5 mM IPTG to induce sucrose production. *A. vinelandii* was grown to exponential phase, washed with PBS buffer, and then inoculated into *S. elongatus* culture 2 days after IPTG induction. The experiment was kept at 30 °C under a constant light intensity at 120 μE m^–2^ s^–1^ with agitation at 120 rpm.

*S. elongatus* growth was monitored in monoculture using a spectrophotometer (750 nm absorbance) and in coculture by flow cytometry. *A. vinelandii* growth was monitored in monoculture using a spectrophotometer (600 nm absorbance) and in coculture using colony-forming units (CFUs) on Burk’s medium agar plates. *A. vinelandii* cells were counted for synthetic cell mixes using CFUs; flow cytometry data (events/μL) were calculated from CFUs using a standard curve (Figure S1).

### Flow Cytometry

Flow cytometric analysis was carried out on an A60-Micro PLUS flow cytometer (Apogee Flow Systems, Hemel Hempstead, UK) equipped with 405, 488, and 561 nm diode lasers. Three photomultiplier tubes were installed to collect small-angle light scatter, medium-angle light scatter, and large-angle light scatter signals. Before sample analysis, the flow cytometer was calibrated using a reference silica beads mix with diameters ranging from 110 to 1300 nm (ApogeeMix, #1493). Cells were measured by using 405-MALS (325 V) and 561 nm orange (500 V) lasers. Data were acquired at a flow rate of 1.5 μL/min with a sample volume of 130 μL under a sheath fluid pressure of 150 mbar and recorded in the Histogram software (Apogee Flow Systems, Hemel Hempstead, UK).

### Protein Extraction

Cell cultures were grown to the exponential phase and harvested by centrifugation at 5000 rpm and 4 °C for 10 min. The pellets were washed by resuspending in 10 mL of PBS buffer and repeating the spin. 500 μL of lysis buffer [2% sodium dodecyl sulfate (SDS; w/v), 40 mM Tris base, pH 8.5, 60 mM dithiothreitol (DTT)] was added to resuspend the pellet. The cells were frozen at −80 °C overnight and quickly thawed at 37 °C to allow for partial cell breakage. 7 μL of 100× Halt protease inhibitor cocktail (Thermo Scientific, Illinois, USA) was added to protect proteins from degradation by endogenous proteases released during protein extraction, and the samples were kept on ice. Complete cell breakage was achieved by vigorous vortex mixing of the samples with 500 μL of 425–600 nm acid-washed glass beads (Sigma-Aldrich, Missouri, USA) 20 times in cycles of mixing for 30 s and cooling on ice for 30 s. Lysates were collected by centrifugation at 13,000 rpm and 4 °C for 10 min.

Crude protein samples were purified using a 2D Clean-Up Kit (GE Health, Buckinghamshire, UK) to remove excess salts, buffers, and other contaminants following the manufacturer’s instructions. Protein concentrations were measured using BradfordUltra reagent (Expedeon, Cambridgeshire, UK), following the manufacturer’s instructions using bovine serum albumin as the protein standard. 2D-purified proteins were used directly for in-solution tryptic digests, as described below. To check the quality of the protein extractions and quantifications, 100 μg of purified proteins was loaded into NuPAGE 12% Bis–Tris Gel (Thermo Scientific, California, USA) running at 200 V for 55 min for protein separation. After SDS-PAGE, gels were washed with distilled water and stained using ReadyBlue Protein Gel Stain (Sigma-Aldrich, Darmstadt, Germany) overnight.

### Sample Preparation

Two types of synthetic mixes were prepared, i.e., protein level mixes and cell level mixes, to assess different quantification methods of coculture. Protein mixes were made by mixing the extracted proteins of *S. elongatus* and *A. vinelandii* at ratios of 100:0, 95:5, 90:10, 75:25, 50:50, 25:75, 10:90, 5:95, and 0:100, named pSA1, pSA2, pSA3, pSA4, pSA5, pSA6, pSA7, pSA8, and pSA9, respectively. Cell mixes were prepared by mixing *S. elongatus* and *A. vinelandii* cells at ratios of 100:0, 95:5, 90:10, 75:25, 50:50, 25:75, 10:90, 5:95, and 0:100, named cSA1, cSA2, cSA3, cSA4, cSA5, cSA6, cSA7, cSA8, and cSA9, respectively. The proteins of mixed cells were extracted using the same method shown above.

### Protein Digestion

Protein lysates were digested using the methods reported by Hitchcock et al.^47^ and Razali et al.^48^ with modifications. Briefly, cleaned-up protein pellets were dissolved in 30 μL of urea buffer (8 M urea/100 mM Tris–HCl pH 8.5/5 mM DTT), followed by water bath sonication to fully suspend them. Protein concentrations were determined using a NanoDrop 2000 spectrophotometer (Thermo Scientific, Delaware, USA) with urea buffer as a blank. 50 μg of protein samples (previously mixed based on protein or cell number) were diluted to 10 μL with urea buffer and incubated at 37 °C for 30 min to reduce the protein. 1.5 μL of 100 mM iodoacetamide was added to the protein solutions and incubated in the dark at room temperature for 30 min. 10 μL MS grade trypsin (Promega, Wisconsin, USA) was added in a 1:50 (w/w) protease:protein ratio to the protein solutions, and the solutions were diluted with 58.5 μL of 50 mM Tris–HCl (pH 8.5)/ 10 mM CaCl_2_ to a final urea concentration of 1 M. The protein solutions were incubated overnight in a 37 °C water bath. Trypsin digestion was terminated by adding formic acid to a final concentration of 1%. Digested peptides were desalted using Bond Elut OMIX C18 tips (Agilent Technologies), following the manufacturer’s instructions, and dried using a SpeedVac.

### Shotgun LC-MS/MS Analysis

Liquid chromatography-tandem mass spectrometry (LC-MS/MS) proteomic analysis was performed following the methods previously reported^49^ with modification. Dried peptide pellets were dissolved in 50 μL of loading buffer, consisting of 3% acetonitrile and 0.1% trifluoroacetic acid in water, and sonicated in a water bath for 3 min for full suspension, after which they were cleared by centrifugation at 13,000 rpm for 2 min. LC-MS/MS was performed and analyzed by nanoflow liquid chromatography (U3000 RSLCnano, Thermo Fisher Scientific, United Kingdom) coupled to a hybrid quadrupole-orbitrap mass spectrometer (Q Exactive HF, Thermo Fisher Scientific, United Kingdom). Peptides were separated on an Easy-Spray C18 column (75 μm × 50 cm) using a 2-step gradient from 3% solvent A (0.1% formic acid in water) to 10% B over 5 min and then to 50% solvent B (0.1% formic acid in 80% acetonitrile) over 75 min at 300 nL min^–1^, 40 °C. The mass spectrometer was programmed for data-dependent acquisition with 10 product ion scans (resolution 30,000, automatic gain control 1 × 10^5^, maximum injection time 60 ms, isolation window 1.2 Th, normalized collision energy 27, and intensity threshold 3.3 × 10^4^) per full MS scan (resolution 120,000, automatic gain control 1 × 10^6^, maximum injection time 60 ms) with a 20 s exclusion time.

### Protein Identification and Quantification

For protein identification of the monoculture and coculture samples, a reference database was created using all protein sequences of *S. elongatus* PCC 7942 (2874 sequences) and *A. vinelandii* DJ (5013 sequences) appended with CscB from *E. coli* and SPS from *Synechocystis* sp. PCC 6803 from Uniprot (https://www.uniprot.org/, Feb 2020), resulting in a final database of 7889 protein sequences. Raw MS data files were processed using MaxQuant (2.0.3.0) and its built-in Andromeda search engine for peptide identification and protein inference.^10^ Default settings were used with search parameters set to include the following modifications: oxidation (M) and acetyl (Protein N-term) (variable); carbamidomethyl (C) (fixed). Peptide-spectrum matches and protein identifications were filtered by using a target-decoy approach at a false discovery rate (FDR) of 1%. Label-free quantification (LFQ) and intensity-based absolute quantification (iBAQ) options were selected.^50^

Proteins were quantified using six metrics based on spectral counts (PSMs, unique peptides, and NSAF) and spectral intensity (intensity, iBAQ intensity, and LFQ intensity) at both the protein level and cell level. PSMs, unique peptides, and intensity, iBAQ intensity, and LFQ intensity values were obtained from MaxQuant analysis. All the values were obtained from MaxQuant output except NSAF, which is equal to the PSMs count divided by protein length.

### Assessment of Physicochemical Characteristics and Shared Peptides

The theoretical *pI* and *M*_w_ of all *S. elongatus* and *A. vinelandii* proteins were assessed by R scripts (Materials S1 and S2) using the proteome sequences obtained from Uniprot (Feb 2020). The hydrophobicity of *S. elongatus* and *A. vinelandii* proteomes was calculated using an R script (Material S3) by GRAVY scores. Theoretically and actually, shared peptides between *S. elongatus* and *A. vinelandii* were compared. A theoretical tryptic digest was performed based on the protein sequences of *S. elongatus* and *A. vinelandii* retrieved from Uniprot (Feb 2020). An R script (Supporting Information Material S4) was prepared to read the protein sequences and theoretically digest them into tryptic peptides by cleaving them after arginine and lysine. Peptides ranging in length from 6 amino acids to 25 amino acids were identified and compared. Measured shared peptides were analyzed by comparison of the resulting peptide sequences of each strain using Hiplot (https://hiplot.com.cn/cloud-tool/drawing-tool/detail/113). The influence of database size was evaluated by searching the *S. elongatus* and *A. vinelandii* coculture mass spectrometry raw data against individual and merged databases.

### Using Modeling to Predict Cell Type Fraction from Quantitative Proteomics

To predict the cellular composition of a mix of *S. elongatus* and *A. vinelandii* from protein identification and quantification data, the proteinGroups file output from MaxQuant was used to train a model. The model registered the LFQ intensity of proteins extracted from synthetic cell mixes of known compositions *S. elongatus* and *A. vinelandii*, with cell ratios ranging from 0 to 100% by steps of 10%. There were three biological replicates for a given cell fraction and three technical replicates for each biological replicate. More ratios were tested to “fill gaps” in the original data set modeled. The following model was trained using the data set corresponding to the three technical replicates for each one of the three biological replicates of synthetic cell mixes for each cell fraction. Briefly, protein identifications that did not belong to *S. elongatus* and *A. vinelandii* were excluded. For each condition, LFQ intensities were normalized across proteins so that the sum of LFQ intensities was one for each experiment. For a given protein, the median of the technical replicates was retained, leaving 33 conditions. A principal component analysis (PCA) was performed to reduce the dimensionality of data, and the first principal component was used as a regressor to predict the cell fraction. The in-sample robustness was tested using a leave-one-out approach, more specifically, by leaving one set of biological replicates aside as a test case, while training was performed on the two other sets of biological replicates.

### Data Availability

The mass spectrometry proteomics data have been deposited to the ProteomeXchange Consortium (http://proteomecentral.proteomexchange.org) via the iProX partner repository^51^ with the data set identifier PXD046627.

## Results and Discussion

### Preliminary Physicochemical Characterization and Bioinformatic Analysis

**Figure 1:**
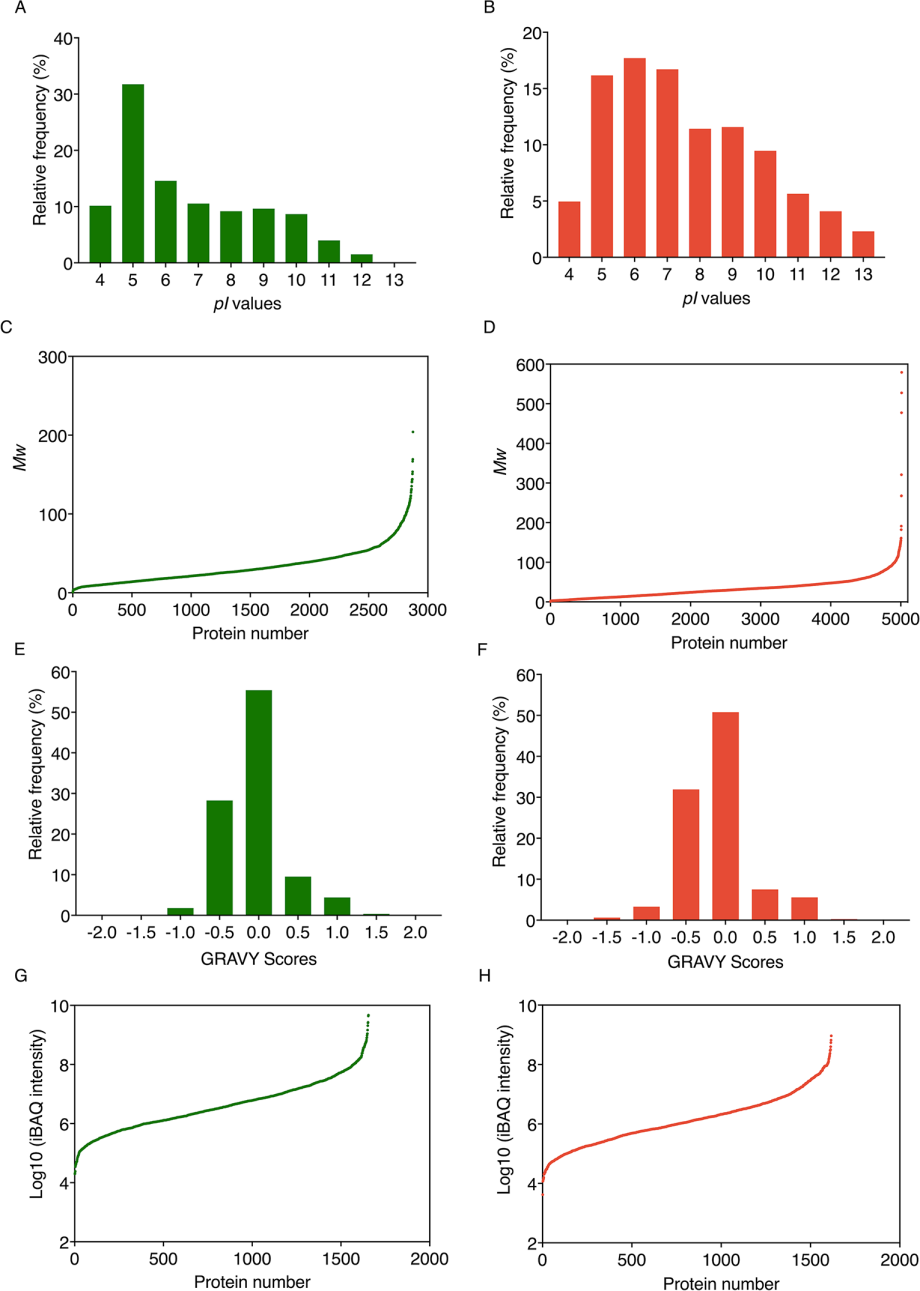
Theoretical *pI*, *M*_w_, hydrophobicity values, and protein dynamic range estimation of the proteins in *S. elongatus* (A, C, E, and G) and *A. vinelandii* (B, D, F, and H) databases. (A) Frequency plot of *pI* of *S. elongatus* database. (B) Frequency plot of *pI* of *A. vinelandii* database. (C) *M*_w_ range of all proteins in *S. elongatus* database. (D) *M*_w_ range of all proteins in *A. vinelandii* database. (E) Frequency plot of GRAVY scores of proteins in *S. elongatus* database. (F) Frequency plot of GRAVY scores of proteins in *A. vinelandii* database. (G) Protein dynamic range estimation of *S. elongatus*. (H) Protein dynamic range estimation of *A. vinelandii*. The iBAQ intensity values for the detected proteins were plotted with log10 iBAQ intensity on the *y*-axis, and proteins were ranked by the iBAQ intensity on the *x*-axis. Theoretical data were calculated using the R script. *pI*: isoelectric point; *M*_w_: molecular weight; GRAVY: grand average of hydropathy.

The physicochemical characteristics of proteins differ between species and can affect protein extraction efficiency and peptide ionization in the mass spectrometer, among other factors, thus influencing protein identification and quantification. Assessing different physicochemical characteristics of the microorganisms in coculture can help decipher whether there are biases in protein identification and quantification between different strains, e.g., different solubilities of proteins from different microorganisms. Protein *pI* is the pH value at which the surface of a molecule carries no net electric charge. Proteins are at their least soluble when the buffer pH is equal to the *pI* value; therefore, this parameter must be considered when optimizing a buffer system to maximize the solubility of proteins. We assessed the theoretical *pI* ranges of *S. elongatus* and *A. vinelandii*, which exhibited similar *pI* ranges of 3.20–13.03 and 3.28–13.16, respectively. The frequency plots (Figure 1A,B) revealed that more than 50% of proteins from both species have *pI* values of 5–7. The *M*_w_ of the proteins in the *S. elongatus* and *A. vinelandii* databases were also assessed. The *M*_w_ of proteins in the *S. elongatus* database ranged from 1.05 to 204.08 kDa (Figure 1C), of which proteins with an *M*_w_ of 1–100 kDa accounted for 98.75% of all proteins. The *M*_w_ range of *A. vinelandii* was from 1.04 to 579.04 kDa (Figure 1D), of which protein *M*_w_ of 1–100 kDa accounted for 98.64%. Our results showed that the proteomes of *S. elongatus* and *A. vinelandii* have similar *M*_w_, although *A. vinelandii* contains several larger proteins.

The hydrophobicity of proteins in the *S. elongatus* and *A. vinelandii* databases was assessed and expressed as the grand average of hydropathy (GRAVY) scores. Negative GRAVY values indicate that the proteins are nonpolar, whereas positive values indicate that the proteins are polar. The frequency plots in Figure 1E and F showed that most proteins have GRAVY scores of −0.5 to 0.5, accounting for 93.25 and 90.19% of *S. elongatus* and *A. vinelandii* protein databases, respectively. Therefore, the difference in physicochemical characteristics between the two species was considered to be minimal based on physicochemical comparisons.

The major obstacle in mass spectrometry-based proteomics is the complexity of the system under study. This challenge becomes even more complex if we further consider all the peptides produced in bottom-up proteomics experiments because ideally, for 10,000 protein-coding genes, hundreds of thousands of analytes should be characterized in order to confidently reconstruct the proteome.^52^ The dynamic range, the range of MS1 peak intensities over which peptides can be detected, is one of the parameters that characterize the complexity of a species proteome.^16,53^ A broad dynamic range, or a very highly abundant protein in one strain, could affect quantification in coculture. The dynamic range of the QExactive HF employed in this study is >5000:1 and has been demonstrated to sequence peptides over 3 orders of magnitude, based on analysis of Hela lysate.^52^ The abundance of detected *S. elongatus* and *A. vinelandii* proteins was quantified by absolute quantification (iBAQ). The scatter plots illustrating the dynamic range with log10 iBAQ intensity show that the dynamic range of both strains covered 5 orders of magnitude (Figure 1G,H), suggesting that the influence of the dynamic range on MS1 detection would be minimal between the two strains. The effective coverage of the proteomes of the two strains was determined as 57.86 and 23.96% for *S. elongatus* and *A. vinelandii*, respectively.

### Proteome Size and Shared Peptides Analysis

**Figure 2:**
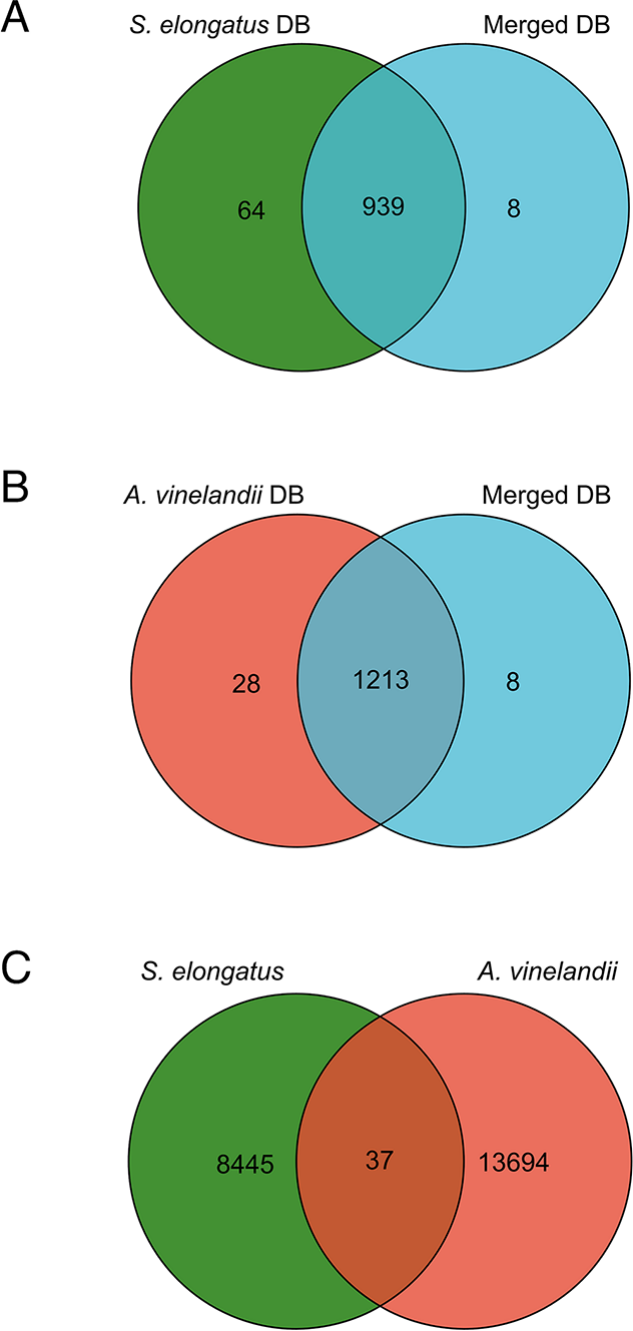
Venn diagrams showing identified protein numbers of *S. elongatus* (A), *A. vinelandii* (B), and shared peptides between *S. elongatus* and *A. vinelandii* (C). (A) Identified protein numbers of *S. elongatus* searching against the individual *S. elongatus* database (green) and the merged *S. elongatus* and *A. vinelandii* database (blue). (B) Identified protein numbers of *A. vinelandii* searching against the individual *A. vinelandii* database (red) and merged *S. elongatus* and *A. vinelandii* database (blue). Venn diagrams were generated using Hiplot. (C) Shared and unique peptides detected between *S. elongatus* and *A. vinelandii*. DB: database.

Database searching is the preferred method for protein identification from digital spectra of the mass-to-charge ratio (*m*/*z*) of protein samples detected by a mass spectrometer.^17^ The quality of the database is one of the main influencing factors in the discovery of proteins present in the sample including the database size. The size of the database determines the computational power required for analysis, the number of peptides identified from the search, and therefore the biological conclusions drawn. To test the effects of database size on protein identification, the spectra of *S. elongatus* and *A. vinelandii* were searched against the individual databases and a larger, merged database. The Venn diagrams illustrate the identified protein numbers of *S. elongatus* (Figure 2A) and *A. vinelandii* (Figure 2B) searched against individual databases and the merged database using proteins with two or more unique peptides. Compared with the individual databases, searching against the merged database reduced the number of identified proteins due to the increased complexity of the merged database content affecting the FDR. This effect was greater on the small proteome database (*S. elongatus*, 2876 sequences) compared to the larger proteome database (*A. vinelandii*, 5013 sequences). However, considering that less than 2% of proteins were not identified when searching against the larger, merged database, the merged database was used in our analyses. However, this should be considered in coculture proteomics workflows, particularly where proteome sizes of coculture members vary more widely.

The LFQ experiment was aimed at identifying proteins in *S. elongatus* and *A. vinelandii* cocultures. Therefore, it is crucial to consider peptides that are shared between the two organisms. For example, peptides from *S. elongatus* might be matched to similar *A. vinelandii* proteins and thus influence protein quantification.^18^ Theoretical shared peptides were calculated, and 0.19% of tryptic peptides of the specified size (8–25 amino acids) were shared between *S. elongatus* PCC 7942 and *A. vinelandii* DJ. Measured shared peptides were also analyzed by comparison of the resulting peptide sequences of each strain, and 0.17% of shared peptides were obtained (Figure 2C), which was similar to the theoretical value. Since this is deemed to be a low number, the assumption made it unlikely that shared peptides will significantly interfere with the protein identification and quantification of each organism. However, we recommend that any shared peptides contributing to protein quantification should be removed so that only unique peptides are used for protein quantifications.

### Proteomics Enables Accurate Quantification at the Protein Level

**Figure 3:**
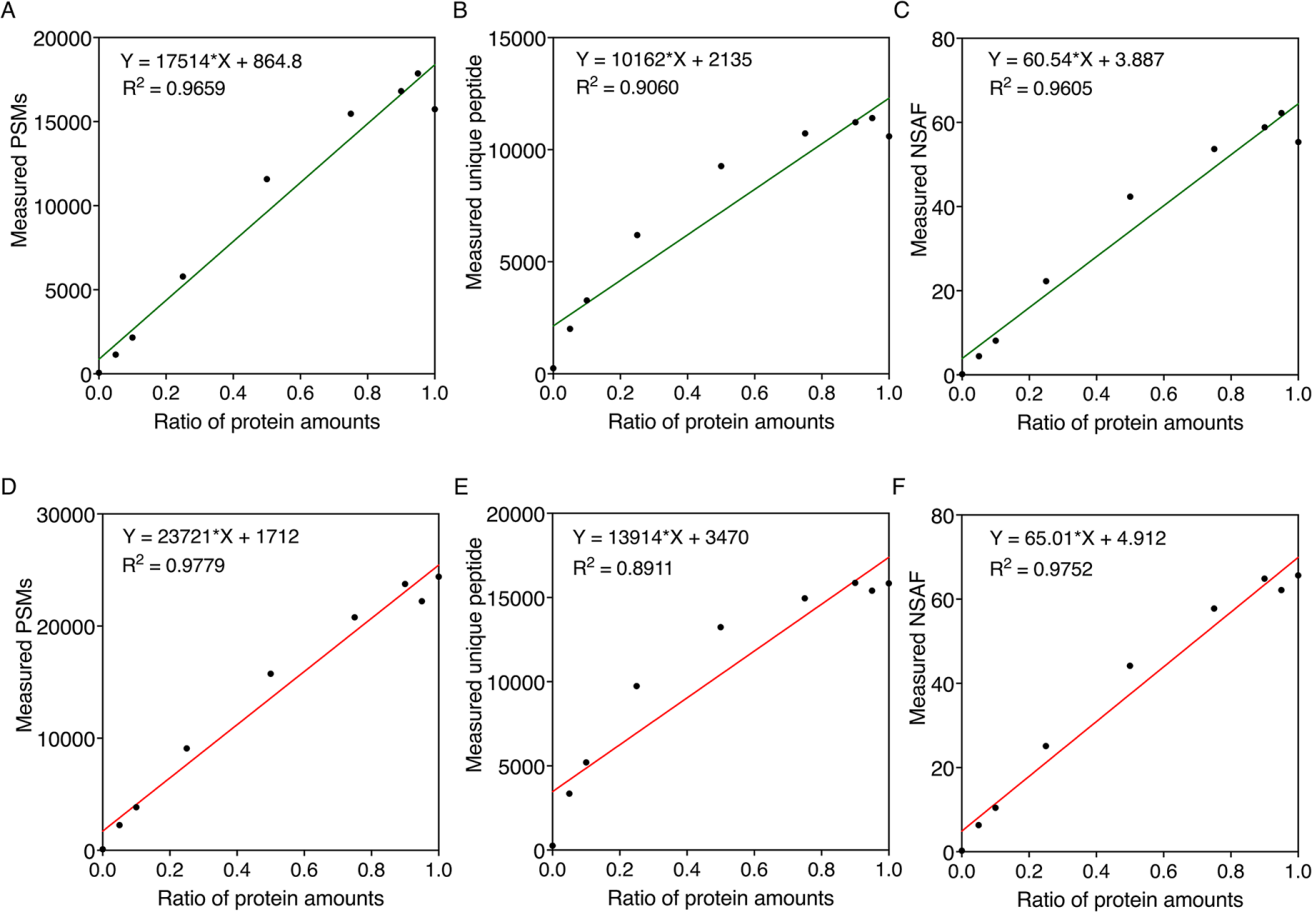
Relationships between absolute protein quantifications calculated using different count-based methods and the ratio of protein amounts in cocultures of *S. elongatus* (top, green) and *A. vinelandii* (bottom, red) at the protein level. I.e., 0.2 refers to 20% of the protein mix being sourced from the specified organism. Protein quantification of *S. elongatus* samples was achieved using three count-based methods: (A) PSMs, (B) unique peptide, and (C) NSAF. Protein quantification of *A. vinelandii* samples was achieved using three count-based methods: (D) PSMs, (E) unique peptide, and (F) NSAF. Linear fittings were analyzed using GraphPad Prism.

**Figure 4:**
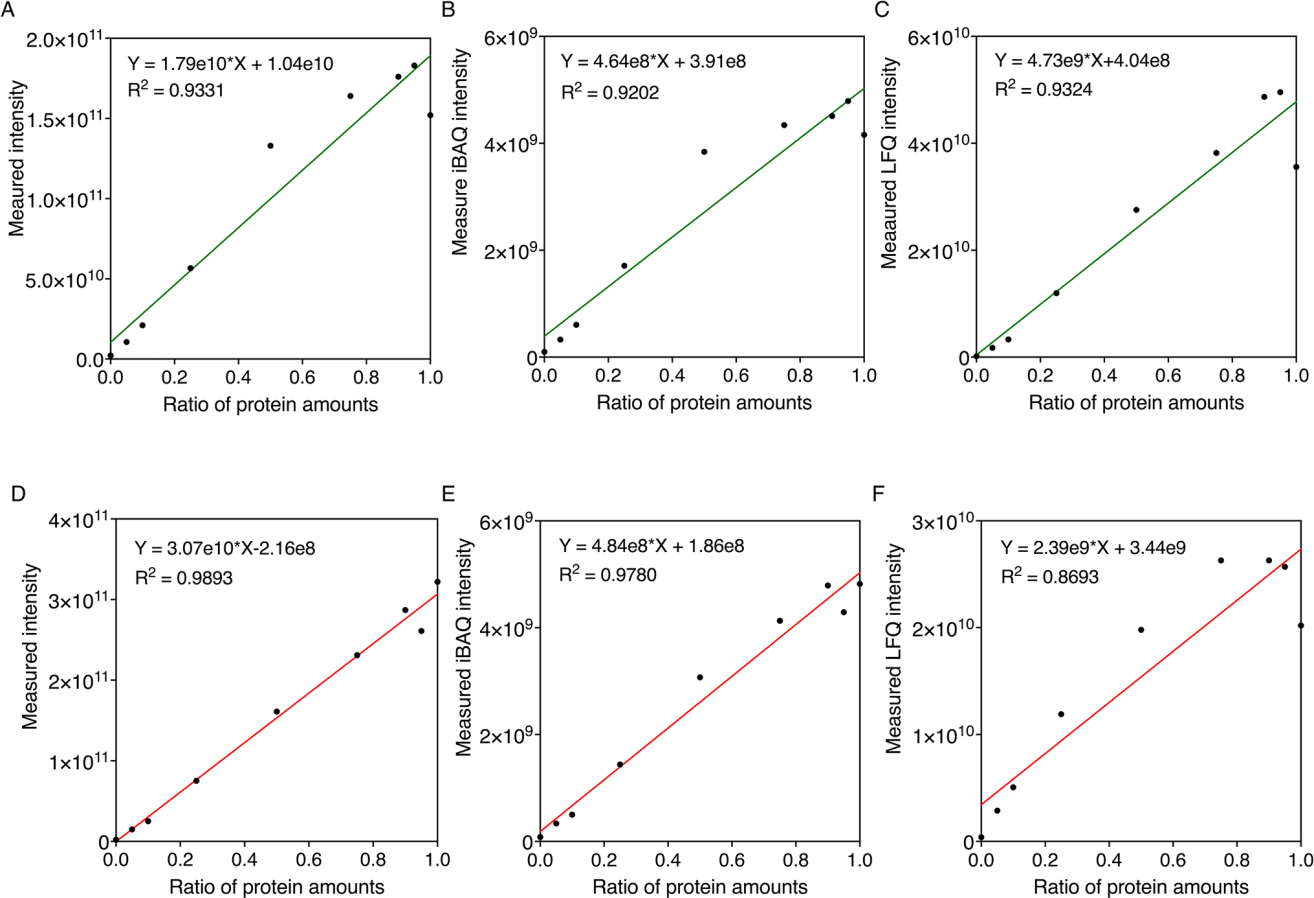
Relationship between absolute protein quantification by different intensity-based methods and ratio of protein amounts of *S. elongatus* (top, green) and *A. vinelandii* (bottom, red) at the protein level. Protein quantification of *S. elongatus* samples was achieved using three intensity-based methods: (A) intensity, (B) iBAQ intensity, and (C) LFQ intensity. Protein quantification of *A. vinelandii* samples was achieved using three intensity-based methods: (A) the intensity, (B) the iBAQ intensity, and (C) the LFQ intensity. Linear fittings were analyzed using GraphPad Prism.

To generate an accurate proteome quantification, it is crucial to test the relationships between different quantification methods and actual protein abundance. We made nine synthetic protein mixes by mixing the extracted proteins of *S. elongatus* and *A. vinelandii* at ratios of 100:0, 95:5, 90:10, 75:25, 50:50, 25:75, 10:90, 5:95, and 0:100 to total amounts of 10 μg (i.e., a 95:5 ratio mix is 9.5 μg *S. elongatus* protein with 0.5 μg *A. vinelandii* protein; Figure S2), and validated different methods for quantifying biomass contribution by HPLC-MS/MS. Three mass spectrometry-based quantification approaches based on the spectral data (i.e., PSMs, unique peptide number, and NSAF) and three approaches based on intensity (i.e., total intensity, iBAQ intensity, and LFQ intensity) were assessed. This is based on the hypothesis that the abundance of a protein is reflected by the proportion of its counts or intensity to the total counts and intensities. We performed linear regression analyses to examine the relationships between absolute protein quantifications calculated using three different count-based methods and protein amounts of *S. elongatus* (Figure 3A–C) and *A. vinelandii* (Figure 3D–F) at the protein level. The linear fittings showed good *R*-squared (*R*^2^) values greater than 0.89, verifying a high level of correlation between protein amounts and spectral counts. We further examined the relationship between protein amounts and absolute protein quantification calculated by different intensity-based methods in *S. elongatus* (Figure 4A–C) and *A. vinelandii* (Figure 4D–F) at the protein level. The *R*^2^ values for each of the linear fittings were again good (>0.9), although *A. vinelandii* absolute protein values quantified by LFQ intensity were marginally lower (*R*^2^ = 0.87), verifying a high level of correlation between protein amounts and spectral intensity.

**Figure 5:**
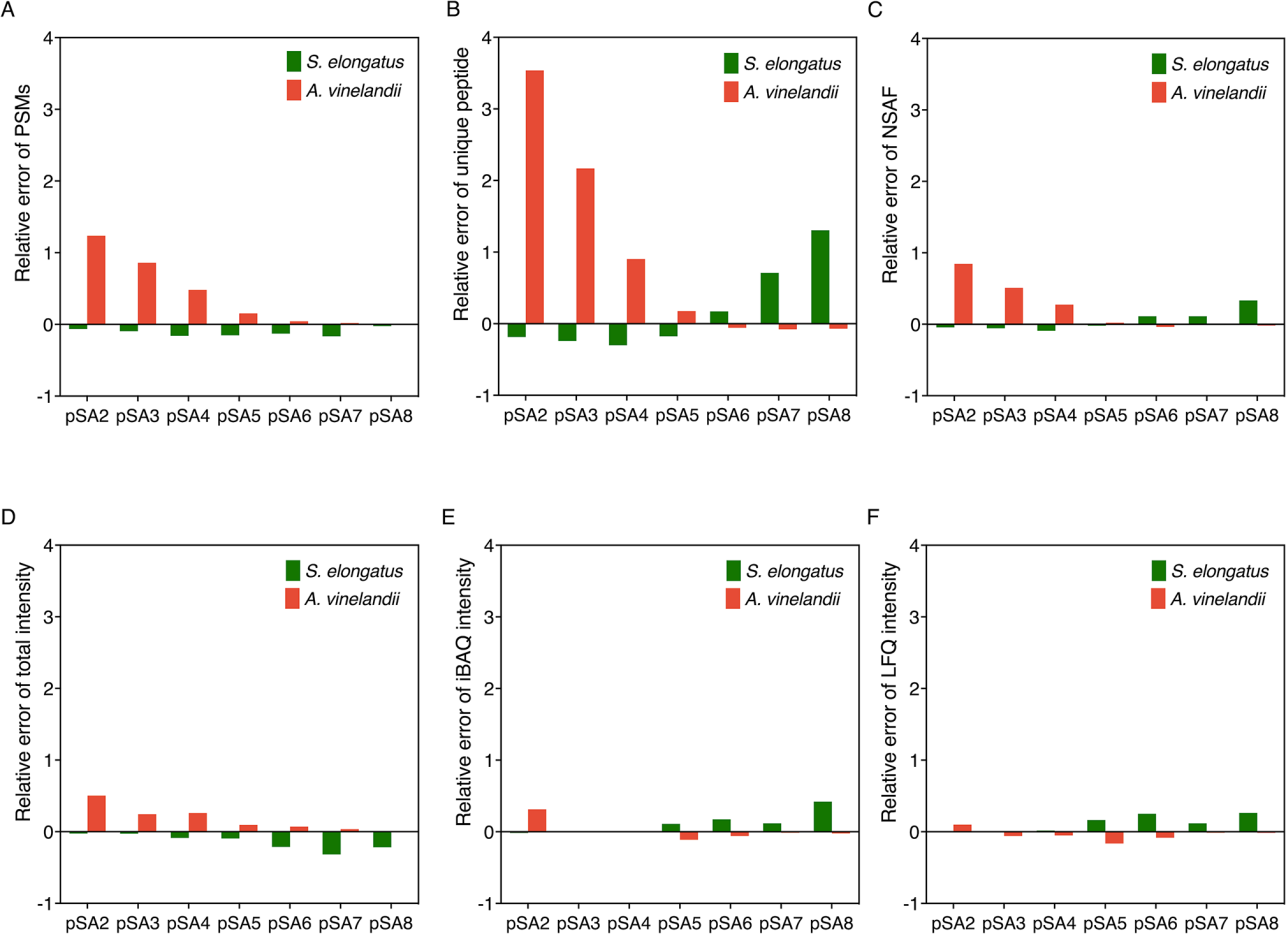
Relative errors of different quantification methods of *S. elongatus* (green) and *A. vinelandii* (red) at the protein level. Spectral count-based quantification: (A) PSMs, (B) unique peptide, and (C) NSAF. Spectral intensity-based quantification: (D) intensity, (E) iBAQ intensity, and (F) LFQ intensity. Relative error = (measured value – real value)/ real value.

Although our results demonstrate good linear relationships between each of the parameters tested and protein amounts at the protein level (Figures 3 and 4), the relative errors of quantification using the intensity-based methods were much smaller than those using count-based methods (Figure 5). This is because spectral counts, which represent the number of MS2 spectra assigned to each protein, include all redundancies of peptide identification, such as charge states, missed cleavages, modifications, and multiple detections of the same peptide resulting from the expired dynamic exclusion.^32^ These redundancies may obscure the relationship between the spectral counts and protein abundance, especially when the machine settings are changed.^35^ We, therefore, concluded that relative label-free quantification calculated using ion intensities enables more accurate proteome quantification in our coculture system.

### Poor Correlation between Protein Quantification and Cell Number for Species with Large Proteomic Databases

**Figure 6:**
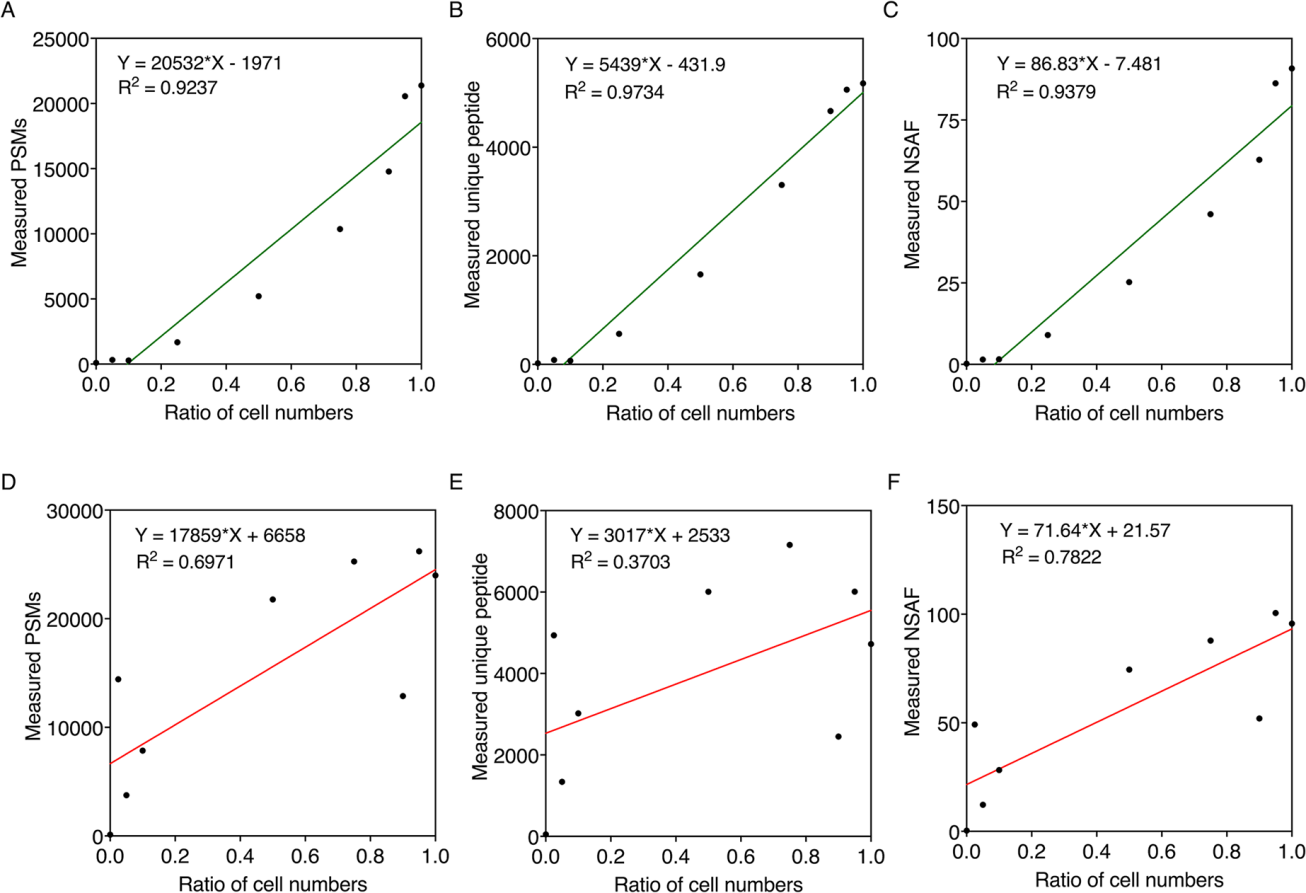
Relationship between absolute protein quantification by different methods and the ratio of cell numbers of *S. elongatus* (top, green) and *A. vinelandii* (bottom, red) mixed at the cell level. Protein quantification of nine different cell ratios of *S. elongatus* samples was achieved using three count-based methods: (A) PSMs, (B) unique peptide, and (C) NSAF. Protein quantification of nine different cell ratios of *A. vinelandii* samples was achieved using three count-based methods: (D) PSMs, (E) unique peptide, and (F) NSAF. Linear fittings were analyzed using GraphPad Prism.

**Figure 7:**
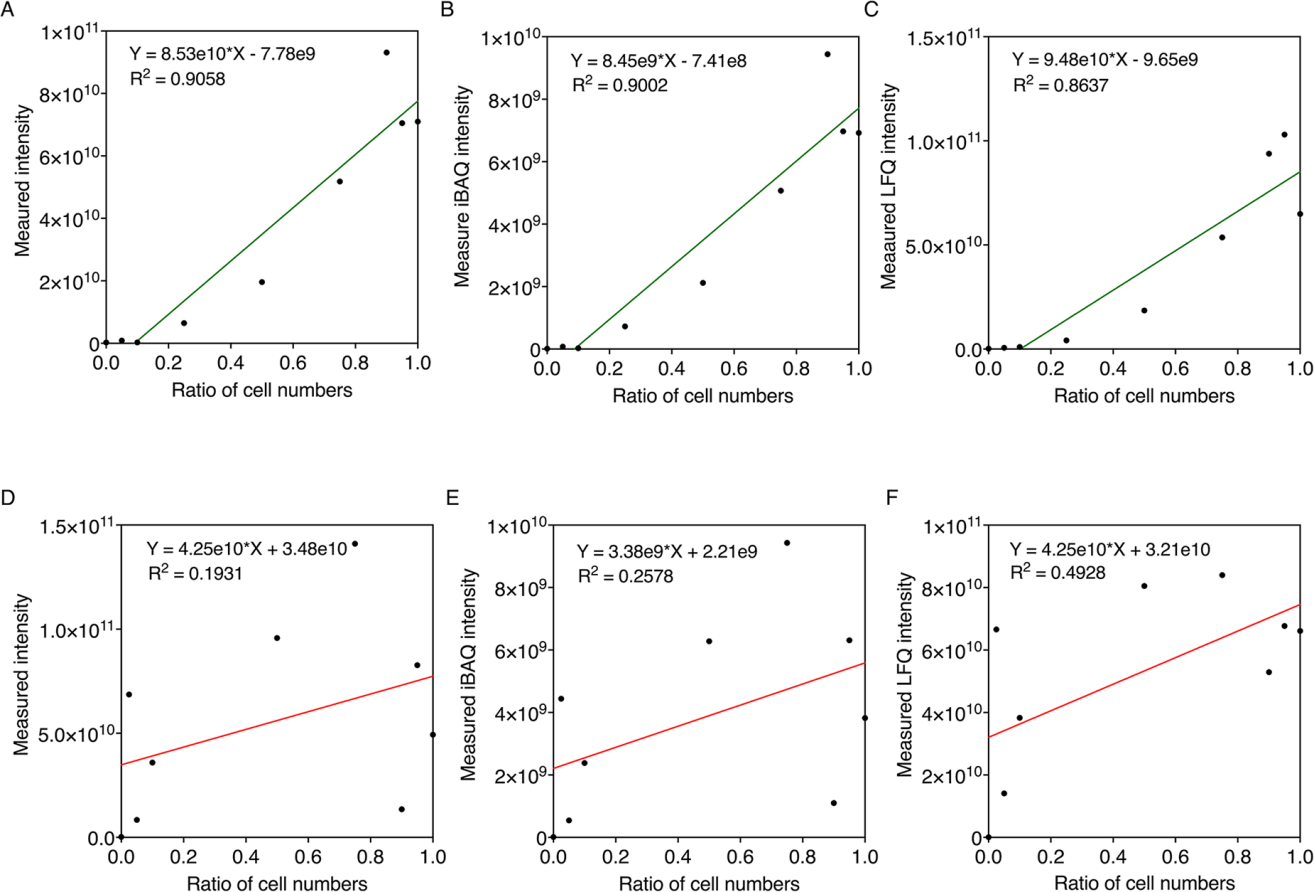
Relationship between absolute protein quantification by different intensity-based methods and the ratio of cell numbers of *S. elongatus* (top, green) and *A. vinelandii* (bottom, red) at the cell level. Protein quantification of *S. elongatus* samples of nine different ratios was achieved using three intensity-based methods: (A) intensity, (B) iBAQ intensity, and (C) LFQ intensity. Protein quantification of *A. vinelandii* samples of nine different ratios was achieved using three intensity-based methods: (A) intensity, (B) iBAQ intensity, and (C) LFQ intensity. Linear fittings were analyzed using GraphPad Prism.

Although mixing proteins extracted from *S. elongatus* and *A. vinelandii* cells in predefined ratios provides insight into protein quantification, showing good linear correlation to spectral counts and intensity, in actual proteomics experiments, cell numbers can vary in ratio in different conditions or over time. Therefore, to investigate the impact of this phenomenon, *S. elongatus* and *A. vinelandii* cells were mixed at nine predetermined cell number ratios of 100:0, 95:5, 90:10, 75:25, 50:50, 25:75, 10:90, 5:95, and 0:100 to assess different quantification methods at the cell level. Fitting plots for *S. elongatus* exhibited good linear relationships, with *R*^2^ values >0.9 between absolute protein quantification by different count-based methods: (PSMs, unique peptide, and NSAF) and cell numbers (Figure 6A–C), whereas poor linear relationships were observed in *A. vinelandii* (Figure 6D–F), revealing weak correlation between cell number and spectral counts. Linear regressions were also generated between absolute protein quantification calculated using different intensity-based methods (intensity, iBAQ intensity, and LFQ intensity) and the cell numbers of *S. elongatus* and *A. vinelandii* samples mixed at predetermined cell number ratios (Figure 7). Very poor correlation between the cell number and spectral intensity was observed for *A. vinelandii* (Figure 7D–F) relative to S. elongatus (Figure 7A–C).

Many factors can reduce the correlation between cell numbers and quantification methods. For example, proteome sizes vary among species, which may lead to differences in protein concentration after extraction. Extraction efficiencies also vary among species, especially when cell sizes differ. Additionally, large proteomic databases can negatively affect the correlation by increasing the time and search complexity, thus reducing the number of peptides identified from the search. These factors make it particularly challenging to predict protein changes when biological states vary in coculture conditions. Therefore, we concluded that to accurately determine protein abundance changes in real biological samples, a normalization method that can minimize the impact of cell number fluctuations among other factors would be required.

### LFQ Intensities Do Not Correlate to Cell Ratios in Cell Mixes

PSMs and intensities have been used to measure proteinaceous biomass contributions of individual species in mock microbial communities composed of 32 species with cell ratios ranging from 0.038 to 21.25%.^11^ We aimed to expand on this further by investigating whether mass spectrometry protein quantification data could also be used to predict cell ratios in cocultures. Our results suggest that LFQ intensity correlates strongly with protein amounts (Figures 4 and 5); therefore, we constructed a model to predict the cell fraction of cell mixes, which we trained using LFQ protein quantification values obtained from synthetic mixes *S. elongatus* and *A. vinelandii* combined in cell:cell ratios varying from 0:100 to 100:0 in increments of 10. A PCA analysis across the samples showed that the first component of this analysis (PC1) carried most of the variance in the data (97%, Figure S3). Our model suggested that only 18 proteins (9 from *S. elongatus* and 9 from *A. vinelandii*) significantly contributed to PC1 (Figure S4) and could therefore be used to predict cell fractions in synthetic mixes. The modeling indicated that, while they can predict cell fractions in synthetic mixes, peptide quantifications (LFQ intensities) will not necessarily generalize very well as we could observe poor correlations to cell mixes in nonsynthetic mixes (Figure S5). A number of reasons can be put forward to explain this. MS typically relies on the comparison of peptide signal intensities between different samples, based on the assumption that observed signal intensities have a linear relationship with peptide abundance. However, many peptides do not display a linear relationship between signal intensity and amount, which is related to observed retention time and its hydrophobicity.^15^ Therefore, it was not accurate to use protein expression quantifications in our synthetic cell mixes to predict the actual cell ratios. Our results so far have emphasized the need for a normalization method able to transform absolute protein quantification data into accurate and biologically meaningful protein abundance values for samples with variable cell ratios.

Thus, different normalization methods were assessed to determine how to factor in the LFQ intensity ratio of each protein and the total protein intensity to generate accurate label-free proteome quantification data for both strains within the microbial coculture. Two approaches were evaluated: one is based on cell number (eq 1) and the other is LFQRatio (eq 2). In eq 1, for each individual protein detected, its LFQ intensity was divided by the number of cells for its respective strain. In eq 2, for each individual protein detected, its LFQ intensity was divided by the sum of all of the protein LFQ intensities for its respective strain.
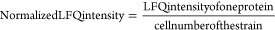
1
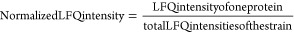
2

### Normalizing Protein Quantification Data by the Cell Number Alone is Unsuitable for Quantifying Proteins in Mixed Cultures

As shown in Figures 6 and 7, we found weak correlations between cell number and spectral counts or spectral intensities in *A. vinelandii*, revealing that protein quantification is not accurate at the cell level. Therefore, we developed a method to adjust protein quantification values, accounting for cell numbers. We used LFQ intensity protein quantification data in our calculation, as the LFQ intensity is a good proxy for protein amount with a low relative error (Figure 5). The LFQ intensities for each protein detected among synthetic cell mixes mixed at known cell ratios were converted to protein amounts per cell by using the LFQ intensity of one strain divided by the total cell number of the strain (eq 1). The values obtained are theoretically the same, as the total protein amount per cell in different cell mixtures of *S. elongatus* or *A. vinelandii* is the same.

**Figure 8:**
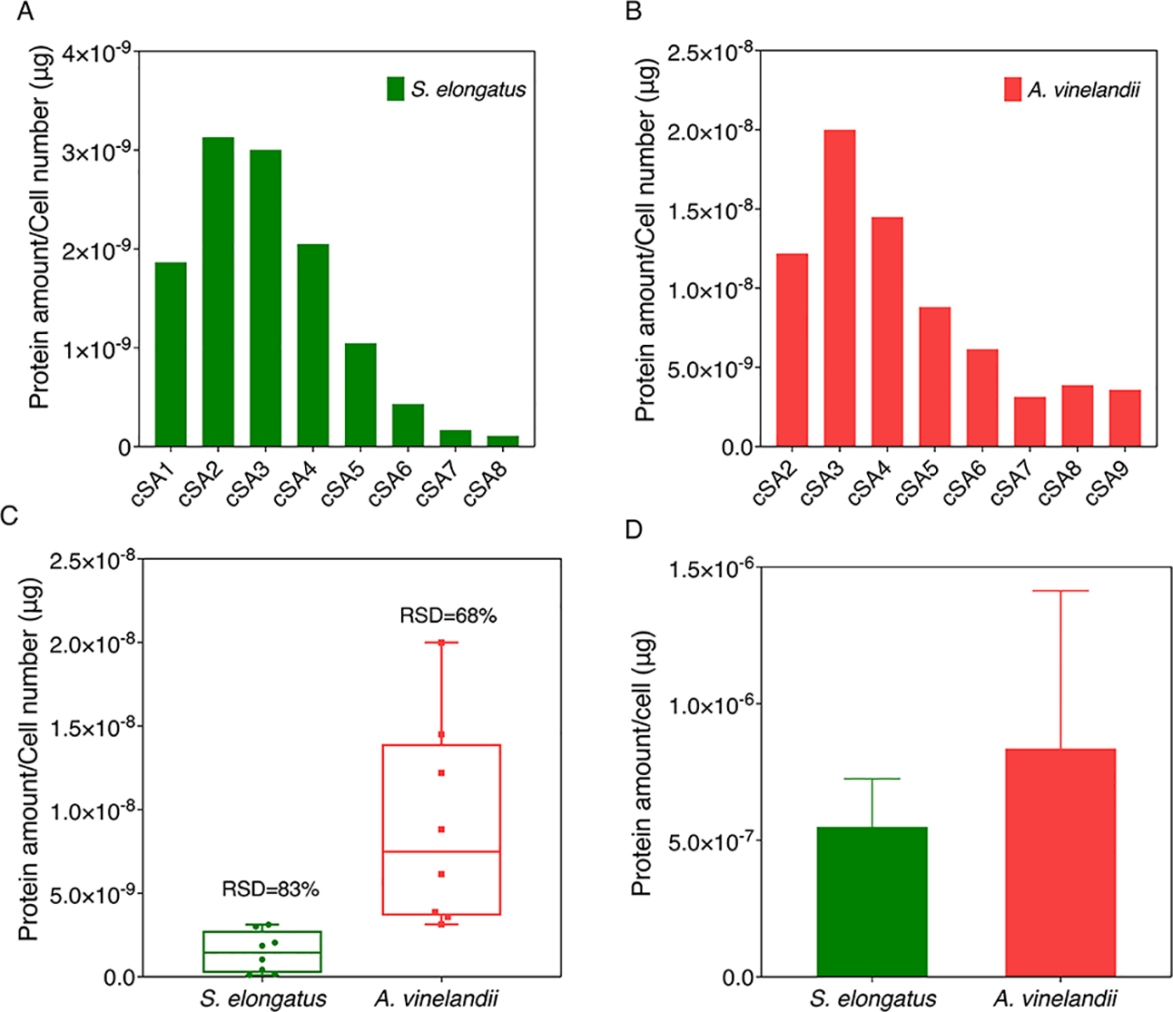
Normalization analysis of proteomic data in cell mixture samples based on the cell number. Normalized LFQ intensities were converted to the protein amount per cell using the linear relationship equations between LFQ intensity and protein amount shown in Figure 3C,F. (A) Protein amount per cell of *S. elongatus* in cSA1 to cSA8 after normalization. (B) Protein amount per cell of *A. vinelandii* in cSA2 to cSA9 after normalization. (C) Distribution of the protein amount per cell with RSD across different cell mixtures of *S. elongatus* (green) and *A. vinelandii* (red) calculated by normalized LFQ intensity. RSD% = SD/Mean. RSD: relative standard deviation. SD: standard deviation. (D) Actual protein amount per cell of *S. elongatus* (green) and *A. vinelandii* (red) with five different cell numbers and two replicates of each.

To validate our method for normalization by the cell number, protein amounts per cell of *S. elongatus* (Figure 8A) and *A. vinelandii* (Figure 8B) were calculated from normalized LFQ intensity using the linear relationship equations between LFQ intensity and protein amount shown in Figure 4C,F. The inferred protein amount per cell varied widely in different cell mixtures, with relative standard deviation (RSD) of 83 and 68% for *S. elongatus* and *A. vinelandii*, respectively (Figure 8C), which was also far from the actual protein amount per cell values of 5.49 × 10^–7^ μg for *S. elongatus* and 8.36 × 10^–7^ μg for *A. vinelandii* (Figure 8D). Differentially expressed proteins (DEPs) were also analyzed using in-browser LFQ-Analyst software (https://analyst-suite.monash-proteomics.cloud.edu.au/apps/lfq-analyst//);^54^ about 66.19% of all proteins were identified as DEPs among all pairwise comparisons. However, to generate the artificial cell mixes, we mixed cells harvested from the same *S. elongatus* and *A. vinelandii* cultures to minimize variation; therefore, we did not expect to see differential expression between proteins in each cell mixture. This verified that the normalization method using the LFQ intensity divided by cell number is not applicable to cell mixtures.

### Development of a Normalization Method for Coculture Proteomics by LFQ Intensity Ratio

To minimize the impact of cell number changes, a normalization method should be developed for coculture proteomics. Considering that the biological states of the *S. elongatus* and *A. vinelandii* cells were the same across the different cell mix samples, we assumed that each protein amount per cell should be the same across different cell mix samples. This means there are no differentially expressed proteins among sample mixtures. Based on this, the protein abundances for each cell mix ratio were normalized using a different method: for each individual protein detected, its LFQ intensity was divided by the sum of all protein LFQ intensities for its respective strain (eq 2), named LFQRatio normalization.

To validate the LFQRatio protein normalization method, the pairwise correlation of LFQRatio normalized results for *S. elongatus* and *A. vinelandii* ratios of 10:90, 20:80, 30:70, 40:60, 50:50, 60:40, 70:30, 80:20, and 90:10 were analyzed, which exhibited good pairwise relationships for the cell mixtures with all correlation coefficients above 0.9 (Figure S6). Differential protein expression was analyzed in the LFQRatio protein normalized cell mixtures, revealing that only 0.05% of proteins were significantly differentially expressed among all pairwise group comparisons. Therefore, we considered our LFQRatio protein normalization approach (eq 2) suitable for normalizing coculture quantitative proteomics data by minimizing the influence of cell number changes on protein quantification.

### Application of LFQRatio Protein Normalization Method Reveals Nutrients Exchange in Synthetic Microbial Cocultures of *S. elongatus* cscB/SPS and *A. vinelandii* △nifL

In order to evaluate the LFQRatio protein normalization method (eq 2), cocultures of *S. elongatus* cscB/SPS and *A. vinelandii* △nifL were cultivated and the growth rate of both strains were characterized together with ammonium and sucrose concetrations in the media. Growth curves and sucrose and ammonium production are shown in Figure S7.

*A. vinelandii* △nifL growth increased over the first 4 days, consuming the media-provided sucrose as its source of carbon. After this time, growth slowed, although an increase in growth was observed after day 8, presumably due to the increased sucrose concentration. As producers of ammonium, the concentration of this nitrogen source increased from 0 to 7.84 mg/L over the same two-day period as *A. vinelandii* △nifL cells were rapidly growing, followed by a steady decrease. The *S. elongatus* cscB/SPS cells rapidly grew for 4 days, coinciding with a reduction in ammonium, as it consumed this as its nitrogen source after being initially supplied with nitrate. From day 4, a steady *S. elongatus* cscB/SPS cell number was maintained until day 14. Based on these growth and nutrient concentration dynamics, biomass samples were collected on day 0, day 4, and day 8 for LC-MS/MS analysis.

**Figure 9:**
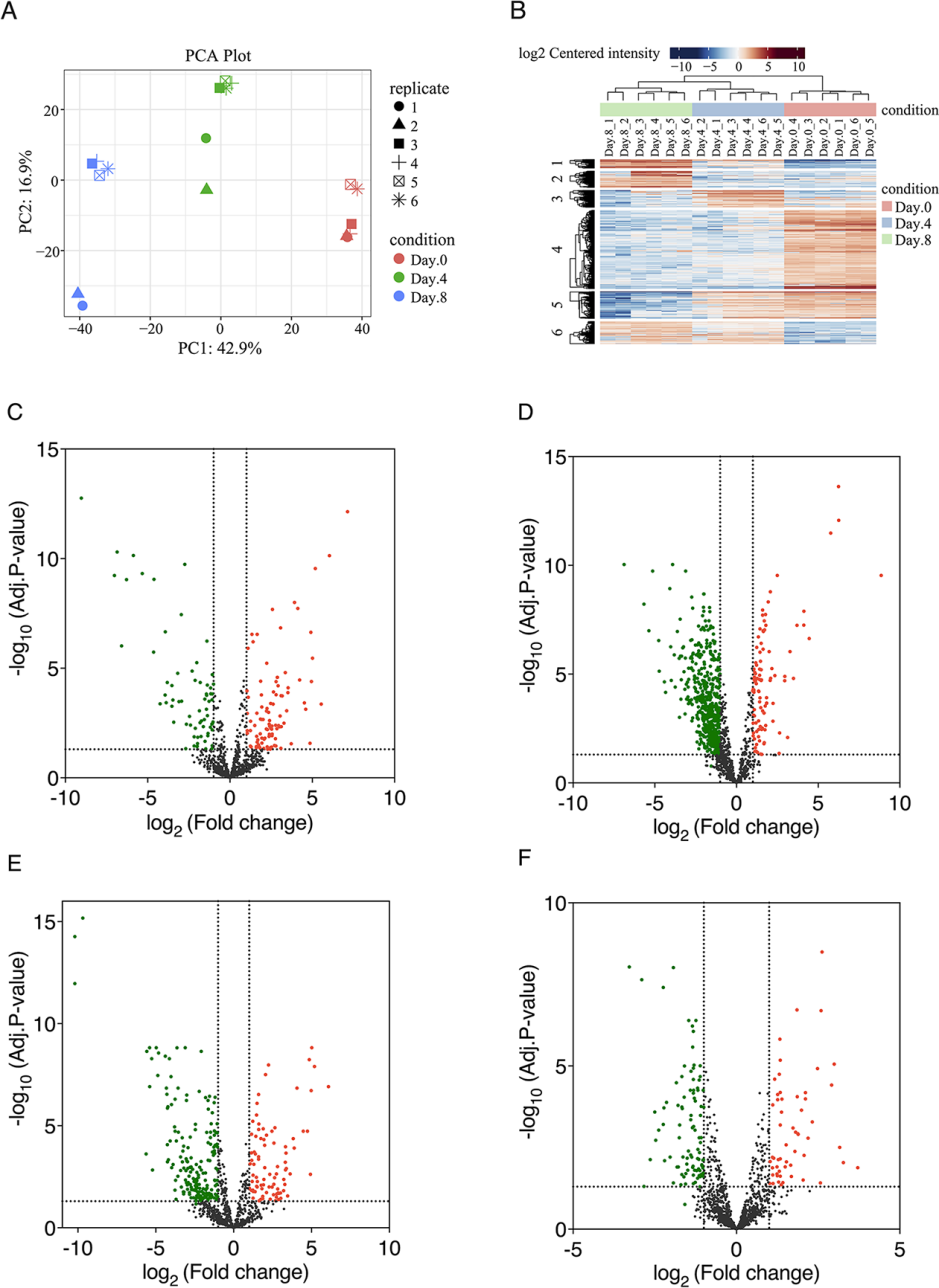
Proteomics data analysis of *S. elongatus* cscB/SPS and *A. vinelandii* △nifL coculture at different time points. (A) PCA plot of individual samples showed distinct clustering of each condition. (B) Heatmap provided an overview of all DEPs (rows) across all samples (Columns) in six clusters. (C) Volcano plot of significantly higher abundance (red, 99) and lower abundance (green, 71) proteins of *A. vinelandii* △nifL on day 4 compared to day 0 after coculture. (D) Volcano plot of significantly higher abundance (red, 100) and lower abundance (green, 520) proteins of *S. elongatus* cscB/SPS on day 4 compared to day 0 after coculture. (E) Volcano plot of significantly higher abundance (red, 88) and lower abundance (green, 188) proteins of *A. vinelandii* △nifL on day 8 compared to day 4 after coculture. (F) Volcano plot of significantly higher abundance (red, 56) and lower abundance (green, 94) proteins of *S. elongatus* cscB/SPS on day 8 compared to day 4 after coculture. The dotted lines represented log2 fold change cutoff of 1 and adjusted *p*-value cutoff of 0.05.

In total, MaxQuant identified 1556 proteins in *S. elongatus* cscB/SPS and 1158 proteins in *A. vinelandii* △nifL. Quantitative results containing protein LFQ intensities were used to generate a list of differentially expressed proteins (DEPs) using LFQ-Analyst. This was undertaken prior to and after normalization using our proposed LFQRatio method. Principle component analysis showed a distinct clustering of biological replicates for each day (Figure 9A). The heatmap provided an overview of all DEPs across all samples, indicating that the proteomes fluctuated with time (Figure 9B). The results revealed 58.48% of proteins differ significantly between samples across all conditions (adjusted *p* < 0.05) (Figure 9C–F).

To determine how the metabolism of *S. elongatus* cscB/SPS and *A. vinelandii* △nifL differed over time, we employed KEGG analysis to assign the function to the differentially expressed proteins and to classify them into specific cellular processes and metabolic pathways. In our normalized data set, from day 0 to day 4, six proteins involved in the photosynthesis pathway exhibited a higher relative abundance of *S. elongatus* cscB/SPS, including photosystem I iron–sulfur center (log_2_FC of 1.53), photosystem I subunit IV (log_2_FC of 1.46), photosystem II reaction center W protein (log_2_FC of 1.07), plastocyanin (log_2_FC of 2.17), ferredoxin (2Fe–2S) (log_2_FC of 1.54), and cytochrome c550 (log_2_FC of 1.42). In addition, glyceraldehyde-3-phosphate dehydrogenase (log_2_FC = 1.59) involved in the carbon fixation pathway also showed higher abundance. These DEPs suggested the promotion of carbon assimilation of *S. elongatus* cscB/SPS, which was evidenced by its increase in the rate of growth over this time period.

Also in *S. elongatus* cscB/SPS, l-glutamine synthetase, which catalyzes the condensation of l-glutamate and ammonia to l-glutamine, showed higher abundance in this time period (log_2_FC of 1.68), while nitrate transport permease and nitrate transport ATP-binding subunits C and D (with log_2_FC of −6.05, −4.5, and −9.1, respectively), involved in membrane transport of nitrate uptake, exhibited significantly lower abundance on day 4 compared to day 0. This was expected as *S. elongatus* cscB/SPS switched from nitrate utilization in the original medium to ammonia use, produced by *A. vinelandii* △nifL. These membrane transport-related proteins did not show differential expression without our LFQRatio normalization.

In the same time period, *A. vinelandii* △nifL cells increased the abundance of sucrose-6-phosphate hydrolase and glucokinase, with log_2_FC of 2.64 and 3.96, respectively. This indicated enhanced uptake of sucrose over this time period, confirmed by a reduction in sucrose concentration in the media as cell numbers increased. Similarly, from day 4 to 8, plastocyanin (log_2_FC of 1.03) and sucrose phosphate synthase (log_2_FC of 1.17) in *S. elongatus* cscB/SPS exhibited a higher relative abundance, indicating the increase of carbon assimilation and sucrose synthesis by the cyanobacteria. l-glutamine synthetase (log_2_FC of 1.43) also showed higher abundance in *S. elongatus* cscB/SPS, suggesting utilization of ammonia produced by *A. vinelandii* △nifL. If normalization via the LFQRatio method is not performed, sucrose phosphate synthase shows lower relative abundance (log_2_FC of −0.773), which conflicts with the increased sucrose concentration (Figure S7B).

These proteomics results of the real synthetic microbial cocultivation reveal the nutrient exchanges between *S. elongatus* cscB/SPS and *A. vinelandii* △nifL, verifying the growth rate dynamics, as well as sucrose and ammonium concentration changes in the media. We also constructed volcano plots to compare the distribution of DEPs between day 4 and day 0, with and without LFQRatio normalization (Figure S8). At day 0, the ratio of *S. elongatus* cscB/SPS and *A. vinelandii* △nifL cells was 84.86 and 15.14%, respectively, and at day 4, the ratio was 30.30 and 70.70%, respectively. These highly different strain compositions would lead to larger differences between data sets without the proposed normalization. As can be seen in the volcano plots, due to the higher number of *A. vinelandii* △nifL cells at day 4, without normalization, the distribution of DEPs is biased toward higher abundance DEPs (Figure S8B). Overall, this helps validate the LFQRatio protein normalization method for quantitative proteomic analysis of microbial cocultures.

## Conclusions

Multiple factors affect the quantification of peptides from individual strains in coculture proteomics, including physicochemical and bioinformatics aspects. Protein quantification was assessed using six different quantification methods, verifying a good linear relationship (*R*^2^ values > 0.9) between the amount of protein and the six selected parameters at the protein level. Different ratios of cell mixes were constructed to mimic the coculture system, which revealed that the correction between cell numbers and quantification parameters can be poor. We, therefore, present a new normalization method, “LFQRatio”, which minimizes the influence of multiple factors such as protein extraction efficiency, different cell numbers, and cultivation conditions on proteome quantification. The overall proteomics workflow can be applied to determine the individual proteome responses of two dynamically different strains cultivated in the same vessel, which can be applied to other biculture or multiculture systems. This will enable researchers to gain new insights into multistrain interactions and their mutual impact on metabolic processes, which were previously unattainable. The LFQRatio protein normalization method can also be used in other species when differences among members in a coculture are minor. However, this model may need to be refined for species with large differences in physicochemical characteristics or with many shared peptides, which represents a difference from the model system described.

## Supporting Information Available

Coculture medium recipe; R script for analyzing *pI* of *S. elongatus* or *A. vinelandii* proteome; R script for analyzing molecular weight of *S. elongatus* or *A. vinelandii* proteome; R script for analyzing hydrophobicity of *S. elongatus* or *A. vinelandii* proteome; R script for analyzing shared peptides between *S. elongatus* and *A. vinelandii*; standard curve of relationship between cell number and CFUs in *A. vinelandii*; SDS-PAGE gel of proteins in sample mixes; principal component analysis (PCA) plot across all replicates, 18 proteins significantly contributing to PC1, correlation of LFQ intensities to cell fractions of *A. vinelandii* and *S. elongatus*, pairwise correlation plots of LFQRatio protein normalized cell mixes, growth and production in coculture, and volcano plots of differentially expressed proteins (DEPs) between day 4 and day 0 with and without LFQRatio normalization (PDF)
