# Supplementary material for: LFQRatio: A Normalization Method to Decipher Quantitative Proteome Changes in Microbial Coculture Systems
Source: J Proteome Res. 2024 Feb 14;23(3):999–1013. doi: 10.1021/acs.jproteome.3c00714 (PMC10913063; doi:10.1021/acs.jproteome.3c00714)
Supplement: Supplementary file 1 — pr3c00714_si_001.pdf [file pr3c00714_si_001.pdf]

Supporting information for

A normalization method to decipher quantitative proteome changes in microbial co-culture systems

Mengxun Shi[1]*, Caroline Evans*[1]*, Josie McQuillan*[1]*, Josselin Noirel*[2]*, Jagroop Pandhal*[1]

[1]Department of Chemical and Biological Engineering, The University of Sheffield, Mappin Street, Sheffield, UK

[2]GBCM Laboratory (EA7528), Conservatoire National des Arts et Métiers, HESAM Université, 2 rue Conté, 75003 Paris, France

Contents

Table S1. Co-culture medium recipe 3

Material S1. R script for analysing *pI* of *S. elongatus* or *A. vinelandii* proteome 3

Material S2. R script for analysing molecular weight of *S. elongatus* or *A. vinelandii* proteome 4

Material S3. R script for analysing hydrophobicity of *S. elongatus* or *A. vinelandii* proteome 4

Material S4. R script for analysing shared peptides between *S. elongatus* and *A. vinelandii* 5

Figure S1: Standard curve of relationship between cell number and CFUs in *A. vinelandii*..6

Figure S2 SDS-PAGE gel of proteins in sample mixes...6

Figure S3. Principal component analysis (PCA) plot across all replicates............................7

Figure S4. Eighteen proteins significantly contributing to PC1 8

Figure S5. Correlation of LFQ intensities to cell fractions of *A. vinelandii* (red) and *S. elongatus* (blue). 8

Figure S6. Pairwise correlation plots of LFQRatio normalized cell mixes. 9

Figure S7. Growth and production in co-culture. 10

Figure S8. Volcano plots of differentially expressed proteins (DEPs) between day 4 and day 0 with and without LFQRatio normalization.. 11

Table S1. Co-culture medium recipe

Ingredients	Amount
sucrose	5 g
$NaNO_3$	0.4 g
$K_2HPO_4 \cdot 3H_2O$	0.826 g
KH_2PO_4	0.2 g
$MgSO_4 \cdot 7H_2O$	0.575 g
$CaCl_2 \cdot 2H_2O$	0.126 g
Citric acid	0.006 g
Ammonium ferric citrate green	0.006 g
$EDTANa_2 \cdot 2H_2O$	0.011 g
Na_2CO_3	0.02 g
$Na_2MoO_4 \cdot 2H_2O$	0.26 g
$FeSO_4 \cdot 7H_2O$	0.015 g
BG11 Trace metal mix	1.0 mL
HEPES	2.383 g
Distilled water	1.0 L

BG11 Trace metal mix

Ingredients	Amount
H_3BO_3	2.86 g
$MnCl_2 \cdot 4H_2O$	1.81 g
$ZnSO_4 \cdot 7H_2O$	0.22 g
$Na_2MoO_4 \cdot 2H_2O$	0.39 g
$CuSO_4 \cdot 5H_2O$	0.08 g
$Co\ (NO_3)_2 \cdot 6H_2O$	0.05g
Distilled water	1.0 L

Material S1. R script for analysing *pI* of *S. elongatus* or *A. vinelandii* proteome

```
library(seqinr)
library(Peptides)
# Load the proteome data
seq = read.fasta("sequence.fasta", seqtype="AA")
# Calculate the pI for the proteome
```

```
pI_scores <- pI(syn, pKscale = "EMBOSS")
pI_score <- mean(pI_scores)
print(pI_score)
```

Material S2. R script for analysing molecular weight of *S. elongatus* or *A. vinelandii* proteome

```
library(seqinr)
library(Peptides)
# Load the proteome data
seq = read.fasta("sequence.fasta", seqtype="AA")
# Calculate the Molecular weight of the proteome
mw_scores <- mw( syn,
    monoisotopic = FALSE,
    avgScale = "expasy",
    label = "none",
    aaShift = NULL
)
mw_score <- mean(mw_scores)
print(mw_score)
```

Material S3. R script for analysing hydrophobicity of *S. elongatus* or *A. vinelandii* proteome

```
library(seqinr)
library(Peptides)
# Load the proteome data
seq = read.fasta("sequence.fasta", seqtype="AA")
# Calculate the hydrophobicity score for the proteome
hydro_scores <- hydrophobicity(seq, scale = "KyteDoolittle")
gravy_score <- mean(hydro_scores)
print(gravy_score)
```

Material S4. R script for analysing shared peptides between *S. elongatus* and *A. vinelandii*

```
library(cleaver)
library(seqinr)
# Load the proteome data
avin = read.fasta("Avin_00010.fasta", seqtype="AA")
syn = read.fasta("Synpcc7942_0001.fasta", seqtype="AA")
avin = sapply(avin, FUN=function (s) paste(s, collapse=""))
syn   = sapply(syn,   FUN=function (s) paste(s, collapse=""))
# Trypsin digestion
avin_pept = unlist(cleave(avin, enzym="trypsin"))
names(avin_pept) = NULL
avin_len = nchar(avin_pept)
avin_pept = avin_pept[8 <= avin_len & avin_len <= 25]
avin_pept = unique(avin_pept)
syn_pept = unlist(cleave(syn, enzym="trypsin"))
names(syn_pept) = NULL
syn_len   = nchar(syn_pept)
syn_pept = syn_pept[8 <= syn_len & syn_len <= 25]
syn_pept = unique(syn_pept)
# Calculate shared peptides
length(intersect(avin_pept, syn_pept))
```

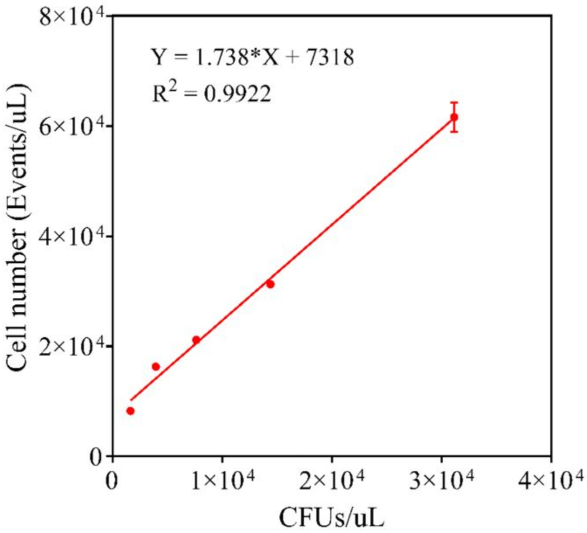

Figure S1: Standard curve of relationship between cell number and CFUs in *A. vinelandii*. Cell number was counted by flow cytometer. CFUs was counted on Burk's agar plate. All data shown were repeated in three replicates. Error bars shown in SD.

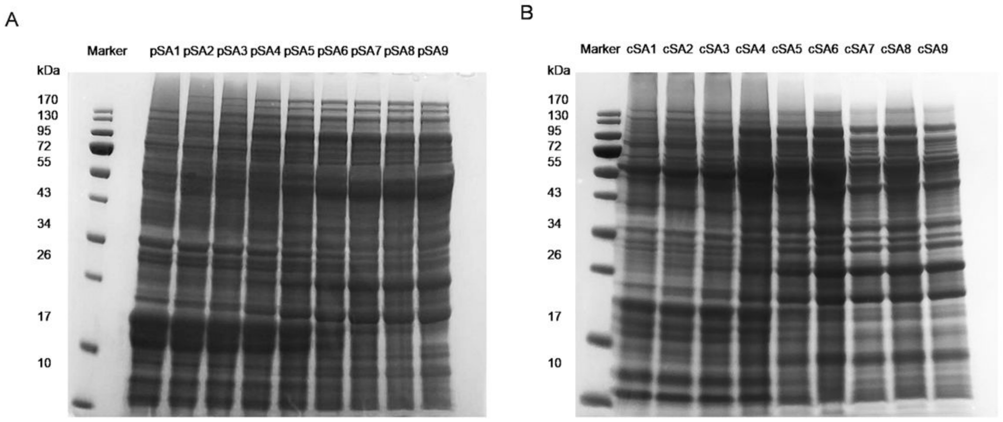

Figure S2 SDS-PAGE gel of proteins in sample mixes. (A) SDS-PAGE gel of different protein mixes; (B) SDS-PAGE gel of different cell mixes. (Edit figure size)

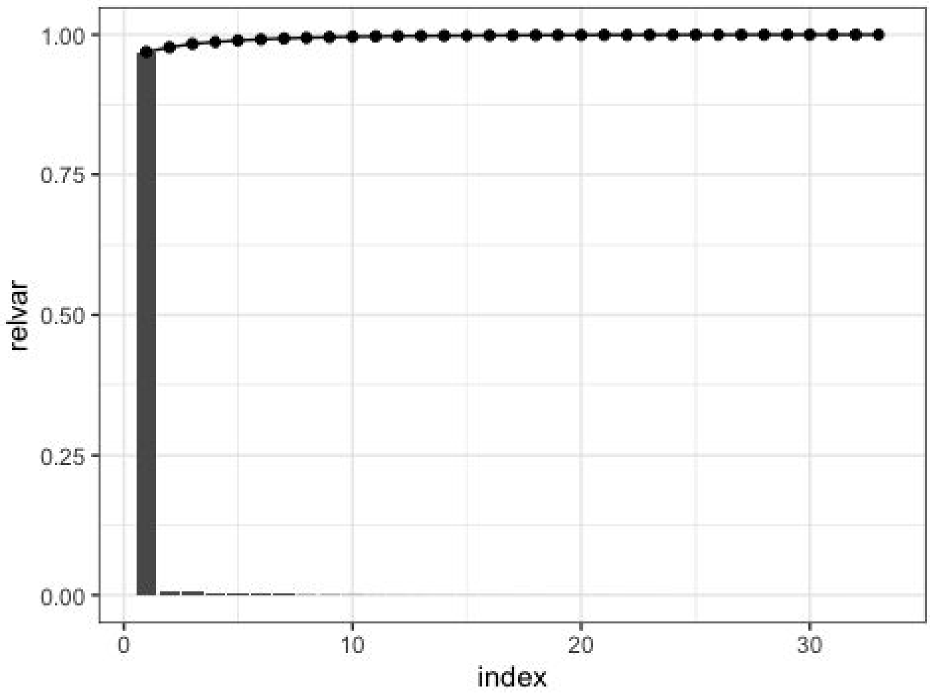

Figure S3. Principal component analysis (PCA) plot across all replicates. The first component of this analysis (PC1) carries most of the variance in the data of 97%. The figure was plotted using R script.

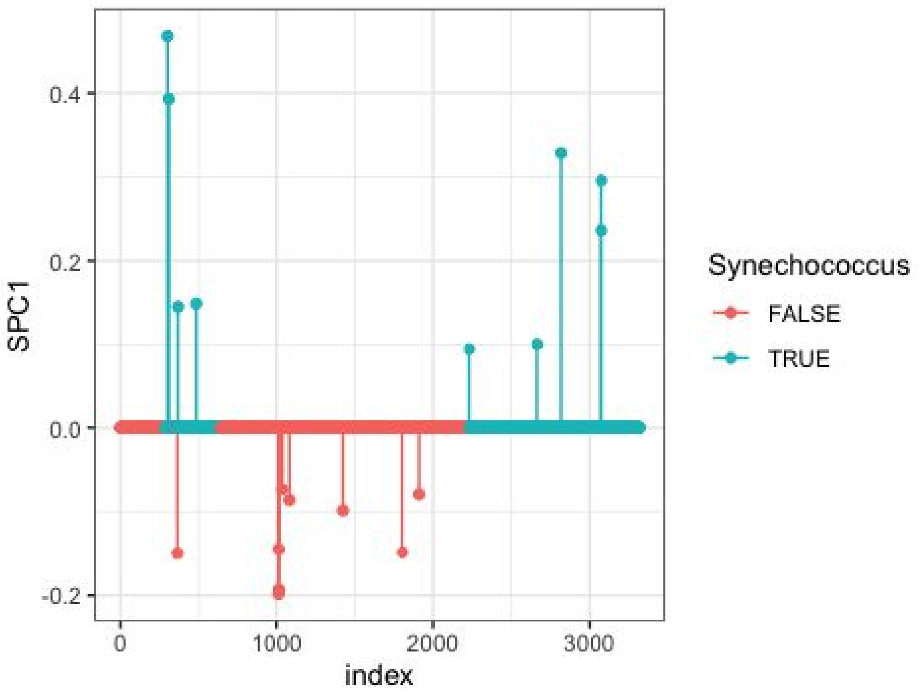

Figure S4. Eighteen proteins significantly contributing to PC1. 9 from *S. elongatus* (blue) and 9 from *A. vinelandii* (red). The figure was plotted using R script.

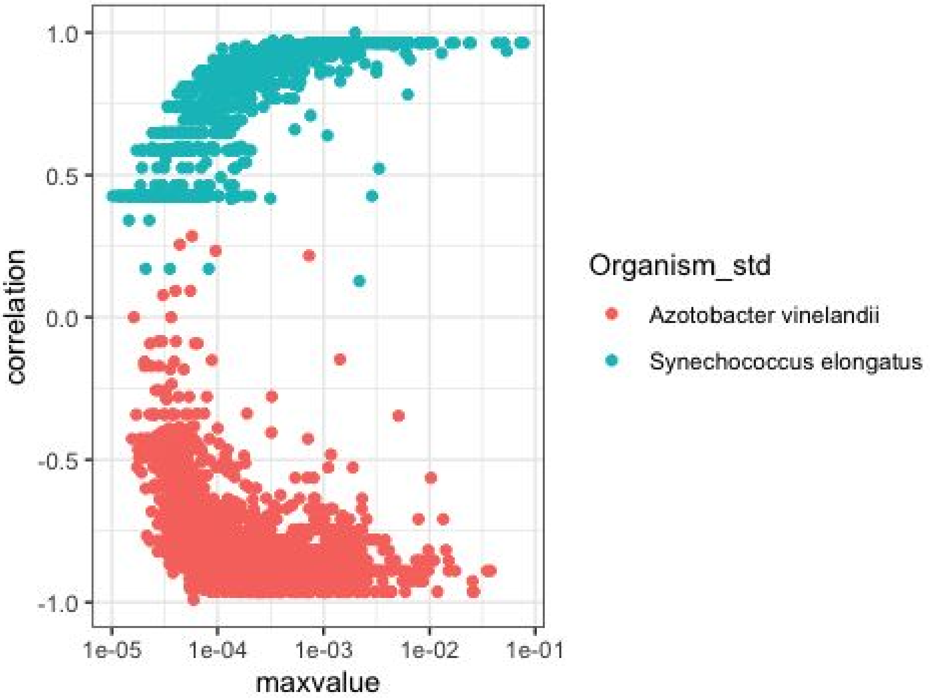

Figure S5. Correlation of LFQ intensities to cell fractions of *A. vinelandii* (red) and *S. elongatus* (blue). The figure was plotted using R script.

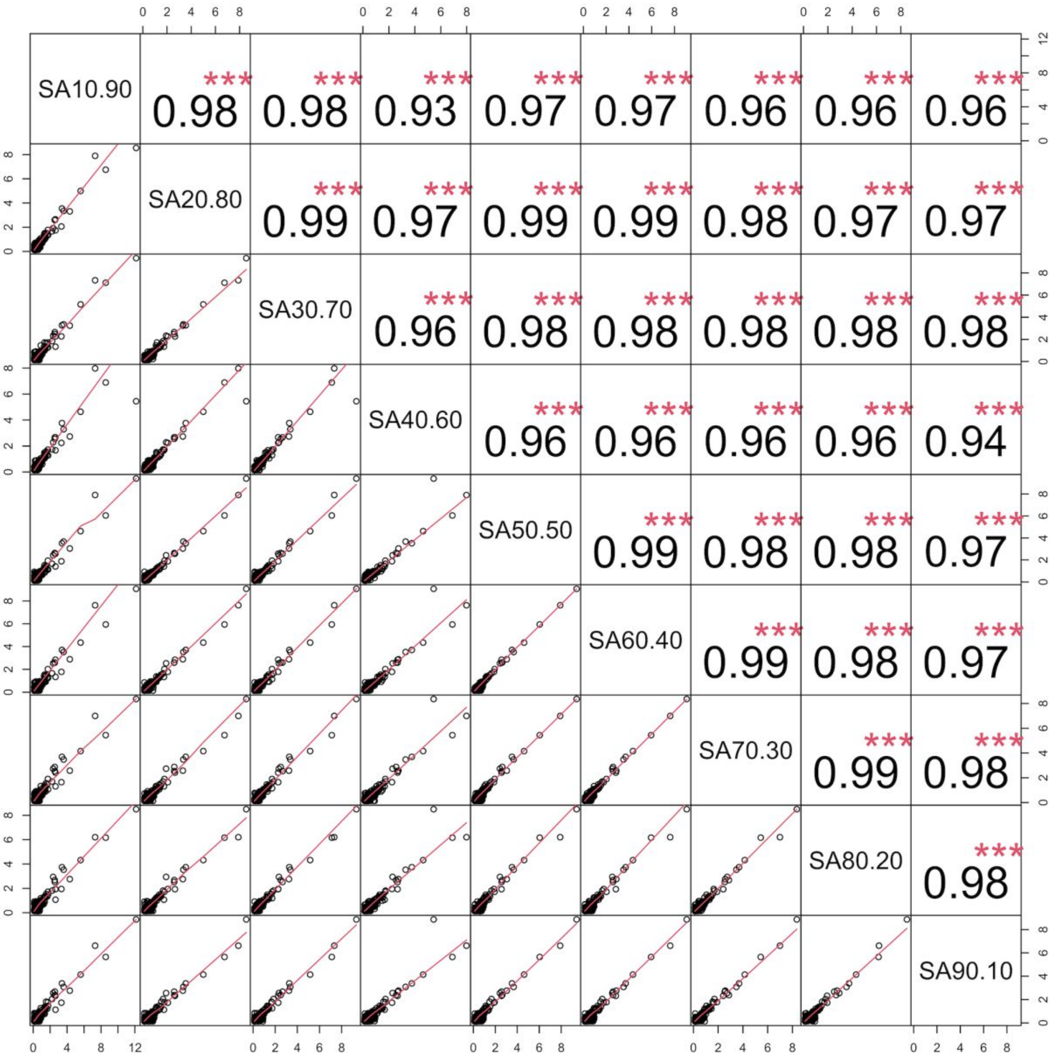

Figure S6. Pairwise correlation plots of LFQRatio normalized cell mixes. The Scatterplot matrix on the lower left part represents the entire dataset of *S. elongatus* and *A. vinelandii* ratios of 10:90, 20:80, 30:70, 40:60, 50:50, 60:40, 70:30, 80:20, and 90:10 without missing values. The red line represents the trend of scatterplots. The data on the upper right part represent correlation coefficients, calculated using the Pearson method. Asterisk represents significance levels (*: p<0.05; **: p<0.01; ***: p<0.001).

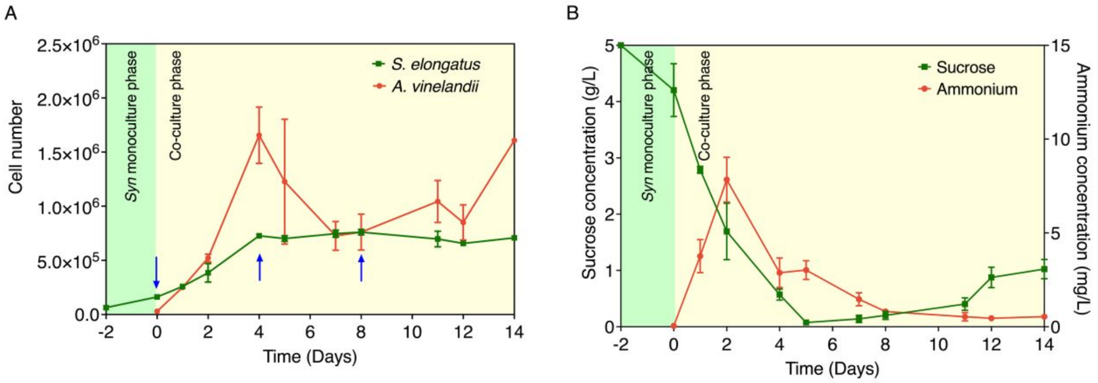

Figure S7. Growth and production in co-culture. (A) Growth curves of *S. elongatus* cscB/SPS (green line) and *A. vinelandii* ΔnifL (red line) in co-culture. *A. vinelandii* ΔnifL was inoculated into *S. elongatus* cscB/SPS culture two days after IPTG induction. (B) Sucrose (green line) and ammonium concentration (red line) in co-culture. Sucrose concentration in the culture was determined using the Sucrose/ D-Glucose Assay Kit (K-SUCGL, Megazyme), according to the manufacturer's instructions. Ammonia concentration was determined using the modified Nessler method. Harvested sample supernatants were diluted to 1 mL with ammonia-free water (VWR Chemicals). Twenty microliters of mineral stabilizer solution (HACH) was added to the samples, then 20 µL of 0.135% (w/v) PVA reagent (Sigma-Aldrich), and 40 µL of Nessler reagent (Sigma-Aldrich). Sampels were vortexed thoroughly and incubated for 10 min at room temperature. Absorbance was measured at 425 nm, and ammonium concentrations were calculated from an ammonium standard curve (ammonia standard solutions; Sigma-Aldrich). Each experiment was repeated in three biological replicates. Error bars represent standard deviation.

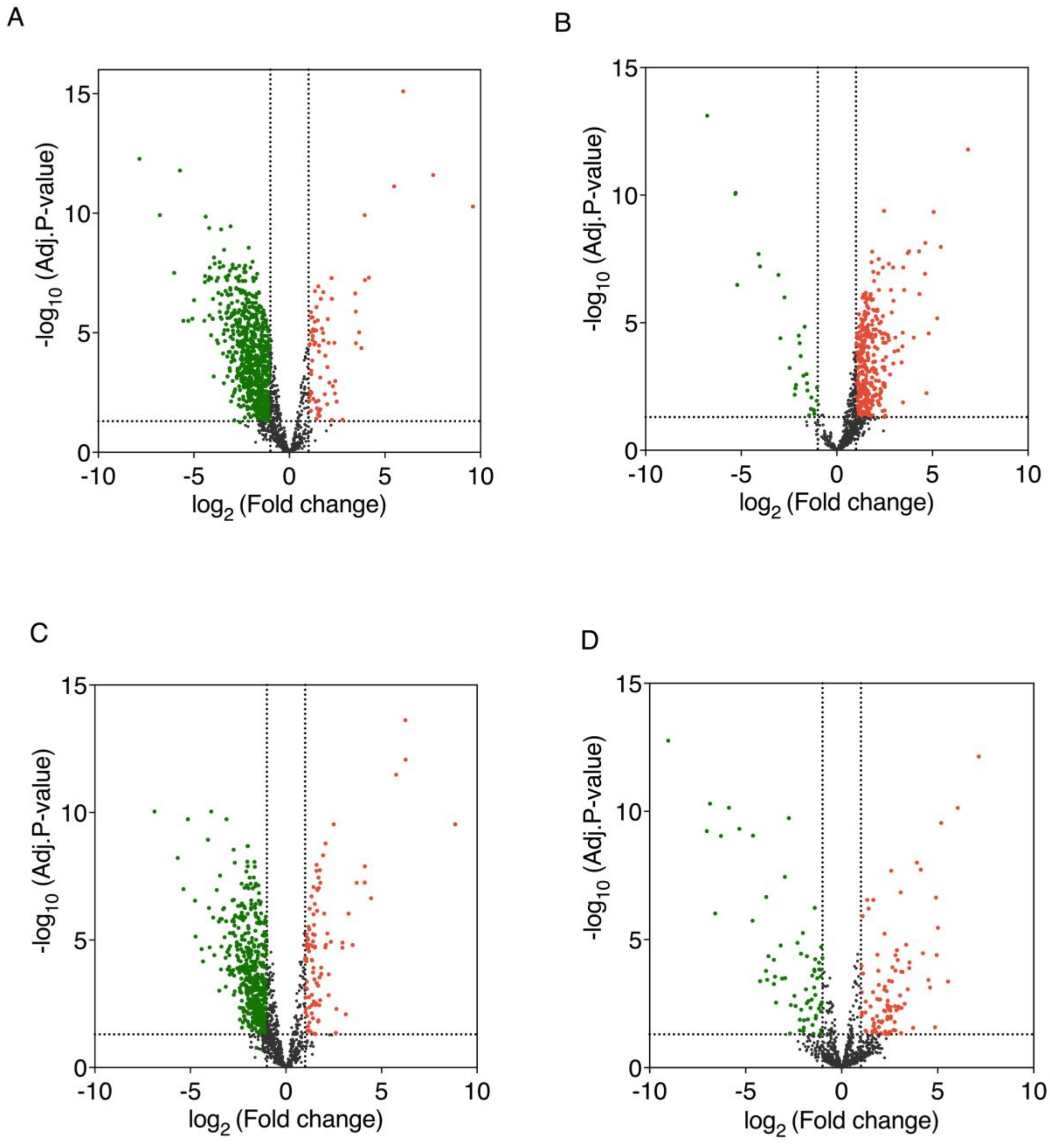

Figure S8. Volcano plots of differentially expressed proteins (DEPs) between day 4 and day 0 with and without LFQRatio normalization. (A) DEPs of *S. elongatus* between day 4 and day 0 without LFQRatio normalization; (B) DEPs of *A. vinelandii* between day 4 and day 0 without LFQRatio normalization; (C) DEPs of *S. elongatus* between day 4 and day 0 with LFQRatio normalization; (D) DEPs of *A. vinelandii* between day 4 and day 0 with LFQRatio normalization.
